# Dravet syndrome: A systematic literature review of the illness burden

**DOI:** 10.1002/epi4.12832

**Published:** 2023-10-11

**Authors:** Adam Strzelczyk, Lieven Lagae, Jo M Wilmshurst, Andreas Brunklaus, Pasquale Striano, Felix Rosenow, Susanne Schubert‐Bast

**Affiliations:** ^1^ Epilepsy Center Frankfurt Rhine‐Main, Center of Neurology and Neurosurgery Goethe‐University and University Hospital Frankfurt Frankfurt am Main Germany; ^2^ LOEWE Center for Personalized and Translational Epilepsy Research (CePTER) Goethe‐University Frankfurt Frankfurt am Main Germany; ^3^ Department of Development and Regeneration University Hospitals KU Leuven Leuven Belgium; ^4^ Department of Paediatric Neurology, Red Cross War Memorial Children's Hospital, Neuroscience Institute University of Cape Town Cape Town South Africa; ^5^ Paediatric Neurosciences Research Group Royal Hospital for Children Glasgow UK; ^6^ School of Health and Wellbeing University of Glasgow Glasgow UK; ^7^ IRCCS ‘G. Gaslini’ Institute Genova Italy; ^8^ Department of Neurosciences, Rehabilitation, Ophthalmology, Genetics, Maternal and Child Health University of Genoa Genova Italy; ^9^ Department of Neuropediatrics Goethe‐University and University Hospital Frankfurt Frankfurt am Main Germany

**Keywords:** caregiver burden, developmental and epileptic encephalopathy, direct costs, health‐related quality of life, indirect costs

## Abstract

We performed a systematic literature review and narrative synthesis according to a pre‐registered protocol (Prospero: CRD42022376561) to identify the evidence associated with the burden of illness in Dravet syndrome (DS), a developmental and epileptic encephalopathy characterized by drug‐resistant epilepsy with neurocognitive and neurobehavioral impairment. We searched MEDLINE, Embase, and APA PsychInfo, Cochrane's database of systematic reviews, and Epistemonikos from inception to June 2022. Non‐interventional studies reporting on epidemiology (incidence, prevalence, and mortality), patient and caregiver health‐related quality of life (HRQoL), direct and indirect costs and healthcare resource utilization were eligible. Two reviewers independently carried out the screening. Pre‐specified data were extracted and a narrative synthesis was conducted. Overall, 49 studies met the inclusion criteria. The incidence varied from 1:15 400–1:40 900, and the prevalence varied from 1.5 per 100 000 to 6.5 per 100 000. Mortality was reported in 3.7%–20.8% of DS patients, most commonly due to sudden unexpected death in epilepsy and status epilepticus. Patient HRQoL, assessed by caregivers, was lower than in non‐DS epilepsy patients; mean scores (0 [worst] to 100/1 [best]) were 62.1 for the Kiddy KINDL/Kid‐KINDL, 46.5–54.7 for the PedsQL and 0.42 for the EQ‐5D‐5L. Caregivers, especially mothers, were severely affected, with impacts on their time, energy, sleep, career, and finances, while siblings were also affected. Symptoms of depression were reported in 47%–70% of caregivers. Mean total direct costs were high across all studies, ranging from $11 048 to $77 914 per patient per year (PPPY), with inpatient admissions being a key cost driver across most studies. Mean costs related to lost productivity were only reported in three publications, ranging from approximately $19 000 to $20 000 PPPY ($17 596 for mothers vs $1564 for fathers). High seizure burden was associated with higher resource utilization, costs and poorer HRQoL. The burden of DS on patients, caregivers, the healthcare system, and society is profound, reflecting the severe nature of the syndrome. Future studies will be able to assess the impact that newly approved therapies have on reducing the burden of DS.

**Key points**

- Impacts on patient HRQoL include the severity of epilepsy, behavior, motor and speech problems, and treatment‐related adverse events.
- The personal, family, social, and professional life of caregivers is impacted, especially mothers, and symptoms of depression are common.
- Direct costs and healthcare resource utilization are substantial, and hospitalizations are the key cost driver.
- A change in working situation for caregivers is common, especially for mothers, leading to high indirect costs.
- A higher seizure burden is associated with greater resource utilization, costs and poorer HRQoL.

## 1 INTRODUCTION

Dravet syndrome (DS) is the prototypical example of a developmental and epileptic encephalopathy (DEE), characterized by the onset of seizures in an otherwise normal infant before the age of 20 months, with neurodevelopment impairments subsequently observed from the second year of life.^1^, ^2^ DS is predominantly caused by pathogenic variants in the *SCNA1* sodium channel gene.^3^, ^4^ Patients typically develop multiple seizure types, most commonly tonic–clonic, hemiclonic, myoclonic, and focal impaired awareness, which can be febrile or afebrile.^5^ Multiple anti‐seizure medications (ASMs) are generally needed to help control the highly drug resistant seizures.^6^, ^7^, ^8^ A prompt diagnosis of DS is important for appropriate treatment, as some ASMs, particularly sodium channel blockers, can exacerbate seizures.^6^, ^7^, ^8^, ^9^

The severity and frequency of seizures tend to decline in adolescence and adulthood, and there is a shift to nocturnal convulsive seizures, the predominant type of seizure in adults.^10^, ^11^, ^12^ However, before that, seizures occur frequently, and can be prolonged.^5^ Despite use of rescue medication, prolonged seizures are often associated with admission to the emergency department, or even to the intensive care unit (ICU) in those who progress to status epilepticus (SE), a situation that can be fatal.^6^, ^13^, ^14^ DS is also associated with premature mortality, in particular, due to Sudden Unexpected Death in Epilepsy (SUDEP) and SE.^12^, ^14^, ^15^, ^16^

Neurodevelopmental disorders are present in the vast majority of DS patients, including intellectual disability, speech impairment, sleep disturbances, motor deficits and gait alterations, and impairments in behavior and psychological functioning.^12^, ^17^, ^18^, ^19^ The latter include symptoms of attention deficit hyperactivity disorder (ADHD) such as hyperactivity and inattention, and autism spectrum disorder (ASD) such as poor social skills and communication abilities.^18^ While the burden of seizures may lessen after the first decade, these cognitive, behavioral, gait, and motor comorbidities continue and increase into adulthood, usually requiring life‐long care.^6^, ^11^, ^20^ Due to the severe and sizeable symptom burden, the quality of life of patients and caregivers is substantially impacted.^12^, ^21^, ^22^, ^23^

We conducted a systematic literature review (SLR) with a narrative synthesis to identify and summarize the evidence associated with the burden of illness in individuals with DS, including the epidemiology (prevalence/incidence and mortality), patient and caregiver health‐related quality of life (HRQoL), direct and indirect costs, and healthcare resource utilization, with the aim of further understanding the impact of DS, as well as identifying potential gaps to direct future research.

## 2 METHODS

### 2.1 Literature search

The SLR was carried out according to the Preferred Reporting Items for Systematic Reviews and Meta‐Analyses (PRISMA) guidelines,^24^ and the protocol was registered with Prospero (CRD42022376561). The following databases were searched from inception to 13th June 2022: MEDLINE, Embase, and APA PsychINFO (all via OVID), Cochrane's database of systematic reviews (CDSR, Wiley), and Epistemonikos. The full search terms, which were developed by an information specialist and checked by two of the authors (AS, SSB), are shown in Appendix S1. In addition, conference proceedings for the years 2019 to June 2022 were searched via Embase. The reference lists of eligible studies and relevant SLRs were searched for additional relevant publications.^25^ The searches were not limited by date (except for conference proceedings) or language and they are reported in web‐only material using a search narrative.^26^

### 2.2 Study selection

The PICOS eligibility criteria (population, intervention, comparator, outcomes and study design) are presented in Table 1. The population were patients with DS (previously known as Severe Myoclonic Epilepsy of Infancy [SMEI]) as defined by the authors; patients with other DEEs were excluded including those that included some patients with DS in a mixed cohort of patients with DEEs. The outcomes were prevalence, incidence and mortality, patient and caregiver HRQoL, direct costs (i.e., costs of resources used in the treatment and management of seizures and DS‐associated comorbidities as defined by the authors), indirect costs (i.e., costs impacting caregivers including changes in employment [lost productivity], out of pocket expenses and/or lost leisure time, as defined by the authors), and healthcare resource utilization (see Appendix S1 for more details of the pre‐specified variables). The focus of this SLR was on studies assessing the overall burden of illness of DS without the impact of an intervention. Therefore, studies assessing interventions, including health economic evaluations, were excluded. Reviews, SLRs and economic evaluations were not eligible, however, their reference lists were searched to ensure all relevant studies were captured. In vitro and in vivo studies, preclinical studies, editorials, and case studies were also not eligible. There were no date restrictions, except for conference proceedings which were limited to the previous 3 years. Articles written in any language were included, although the search terms were only in English as it was expected that all non‐English language publications would include an English language abstract.

**TABLE 1:** PICOS criteria for inclusion and exclusion of studies.

PICOS	Inclusion	Exclusion
Population	Patients with Dravet syndrome (DS)/Severe Myoclonic Epilepsy of Infancy (SMEI)^2^ as defined by the authors	Other DEEs
Intervention	Studies assessing the overall burden of illness of DS without the impact of an intervention	Studies assessing the impact of interventions^a^
Comparator	N/A	–
Outcomes	At least one of the following outcomes is a primary aim: Prevalence/incidence Economic burden ◦ Direct and indirect costs Resource use Patient and caregiver HRQoL (quantitative and qualitative assessments) Mortality	–
Study designs/publication type	Publications reporting original data, including: Healthcare insurance claims data Chart reviews Registry studies Electronic healthcare databases studies Epidemiologic surveys Observational studies	In vitro and in vivo studies Preclinical studies Case reports and case series Clinical studies reporting only efficacy and safety data Economic evaluations reporting on specific interventions^b^ Reviews,^c^ SLRs,^c^ comments, letters, editorials and press releases
Limits	Searches of conferences in Embase were limited to the previous 3 years (conferences 2019‐current)^d^	None

Abbreviations: DEE, developmental and epileptic encephalopathy, HRQoL, health‐related quality of life; N/A, non‐applicable; SLR, systematic literature review.

^a^ The focus of this SLR is on the overall burden of DS rather than the impact of specific interventions.

^b^ Economic evaluations reporting on specific interventions without any original data on e.g HRQoL (utilities) and healthcare resource utilization were excluded, however, the reference lists were searched so that all relevant studies were identified.

^c^ Reviews and SLRs were excluded from final inclusion but the reference lists were searched so that all relevant studies were identified.

^d^ Conference abstracts ≤3 years were included to provide the most up‐to‐date evidence; conference abstracts with a cut‐off of >3 years were excluded, allowing acceptable time for studies of sufficient quality to be published in peer‐review journals.

The online systematic review program Covidence (https://www.covidence.org/) was used to manage and screen the publications identified via database searches. Two reviewers independently screened all articles for inclusion according to the PICOS criteria. Initially, articles that were not relevant according to the PICOS criteria were excluded by screening the title and abstract (primary screening). For secondary screening, full publications were obtained and the full text was evaluated. The reasons for exclusion at secondary screening according to the PICOS criteria were recorded. Any conflicts and uncertainties regarding the inclusion of studies were discussed between the two reviewers and a consensus was reached.

### 2.3 Data extraction and synthesis

Data were extracted into tables in MS word by one reviewer and the data were checked by another reviewer. The pre‐specified variables are presented in Appendix S1. The synthesis of the data was performed for each outcome (epidemiology, patient and caregiver HRQoL, direct and indirect costs and healthcare resource utilization) in text and tables, and where appropriate using graphs. It was anticipated that the studies identified in this review would be diverse with regard to study design, definition of DS, size of the populations, the ages of the patients, and the length of the follow‐up period, and therefore the synthesis of the data was planned to be narrative in nature, without any statistical analyses/comparisons (e.g., meta‐analyses). Potential gaps in the evidence were identified and discussed. Costs were extracted and are described as reported by the authors. Summaries of the costs are presented in US dollars ($), with costs in Euros (€) converted at a rate of €1.00 = $1.00 (Google Finance 30th August 2022), that is, US dollars and Euros were equivalent at the time of writing the narrative synthesis.

### 2.4 Quality assessment

For prevalence studies we used a tool developed by Hoy et al^27^ to conduct quality assessments (risk of bias); for cost of illness studies we used a tool from the British Medical Journal Checklist for economic submissions,^28^ adapted by Molinier et al^29^; for HRQoL qualitative and quantitative studies we used the Critical Appraisal Skills Programme (CASP; https://casp‐uk.net/), adapted by Gallop et al.^21^

## 3 RESULTS

### 3.1 Search results

**FIGURE 1:**
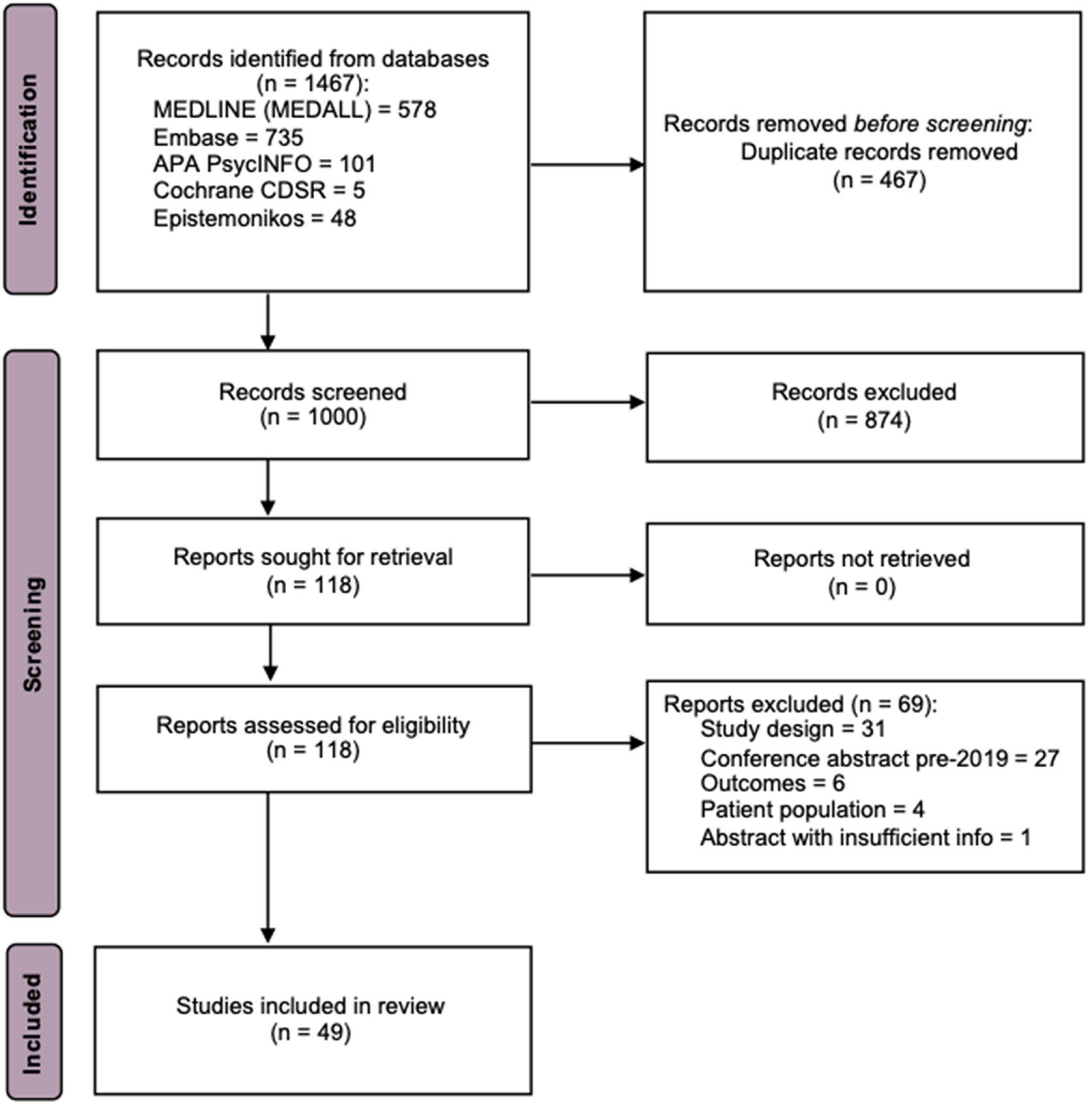
PRISMA flow diagram. Adapted from Page et al.^124^

Overall, 1467 publications were identified from the electronic database searches. After removal of duplicates (n = 467), 1000 publications were screened based on abstract and title (primary screening), and 118 full‐text articles were retrieved for secondary screening. Of these, 49 met the eligibility criteria (Figure 1; Table S1). Epidemiology, HRQoL, costs (direct and indirect) and resource data are presented in Tables S2–S5 (Appendix S2). The results of the quality assessments/risk of bias are presented in Tables S6–S9 (Appendix S2).

### 3.2 Epidemiology: Incidence, prevalence and mortality

Overall, 10 studies were identified that reported on incidence (seven studies^30^, ^31^, ^32^, ^33^, ^34^, ^35^, ^36^) and/or prevalence (five studies^31^, ^34^, ^37^, ^38^, ^39^) (Table S2). The studies differed in their publication year (1990–2022 for incidence and 2015–2022 for prevalence), country (2 from the US,^33^, ^35^ and 5 from across Europe for incidence^30^, ^31^, ^32^, ^34^, ^36^ and 1 from the US,^37^ and 4 from across Europe for prevalence^31^, ^34^, ^38^, ^39^), duration of follow‐up, and patient population including the diagnostic criteria, average age and population size.

The incidence ranged from 1:15 400 to 1:40 900 (1:15 400 to 1:40 000 in studies exclusively in children,^30^, ^31^, ^33^, ^34^, ^35^, ^36^ and 1:40 900 in one study that included both children and adults^32^). The prevalence ranged from 1.5 per 100 000 to 6.5 per 100 000.^31^, ^34^, ^37^, ^38^, ^39^ The risk of bias was low in seven studies and moderate in three studies (Table S6).

Mortality, reported in eight studies (nine publications),^14^, ^15^, ^16^, ^31^, ^32^, ^39^, ^40^, ^41^, ^42^ occurred in 3.7%‐20.8% of DS patients, with one study reporting a mortality rate of 15.8 per 1000 person‐years.^14^ Mortality was significantly higher in DS patients than in age‐ and sex‐matched controls (people without DEEs) identified from the same healthcare insurance claims database over an equal observation time (11.9% vs 1.2%, *P* < 0.001).^39^ The most common reasons for death across all studies were SUDEP followed by SE.^14^, ^16^, ^31^, ^32^, ^40^, ^41^, ^42^

### 3.3 Health‐related quality of life

Overall, we identified 23 publications reporting on patients' (n = 13^43^, ^44^, ^45^, ^46^, ^47^, ^48^, ^49^, ^50^, ^51^, ^52^, ^53^, ^54^, ^55^, ^56^) and/or caregivers' (n = 16^47^, ^48^, ^50^, ^51^, ^52^, ^53^, ^54^, ^56^, ^57^, ^58^, ^59^, ^60^, ^61^, ^62^, ^63^, ^64^, ^65^) HRQoL (Table S3). The quality was assessed to be grade 2 (moderate‐high methodological and reporting quality) in all the qualitative studies (Table S7) and grade 1 (highest methodological and reporting quality) or grade 2 in the quantitative studies (Table S8).

**FIGURE 2:**
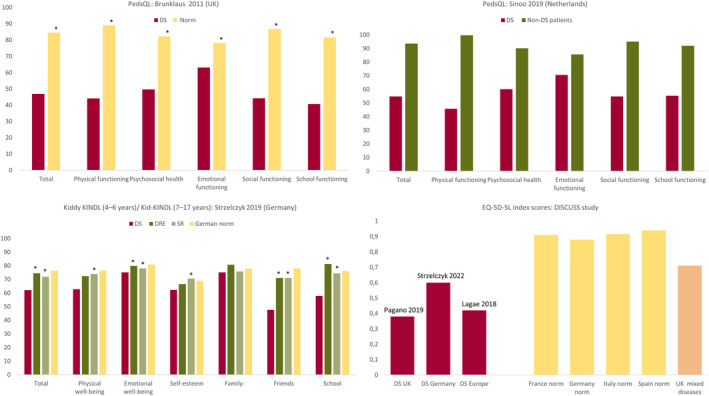
HRQoL in patients with DS. Data are mean values. Higher values = better HRQoL. EQ‐5D‐5L population norm data are from Azzi 2020 for France,^125^ Grochtdreis 2019 for Germany,^126^ Scalone 2015 for Italy,^127^ and Hernandez 2018 for Spain.^128^ EQ‐5D‐5L population norm data are not available for the UK/England and data from a mixed disease population was used instead (Richardson^129^). *Statistically significant difference versus DS (where analyzed). Adapted from Brunklaus,^44^ Sinoo,^51^ Strzelczyk,^54^ Pagano,^48^ Strzelczyk,^56^ and Lagae.^46^

Using caregiver proxy, patient HRQoL was evaluated quantitatively using a variety of tools including PedsQL and Kiddy KINDL (4–6 years)/Kid‐KINDL (7–17 years) (Table S3 and Figure 2). Across four studies (one from the UK,^44^ two from the Netherlands,^45^, ^51^ and one from Poland^49^) mean total PedsQL scores ranged from 46.5 to 54.7, lower than published values from both the general population^44^ and non‐DS epilepsy cohorts^45^, ^51^ (Table S3 and Figure 2). As well as total scores, the scores for the PedsQL domains (physical, psychosocial, emotional, social and school functioning domains) were lower in DS compared to UK norms (statistically significant differences),^44^ and a non‐DS epilepsy cohort in the Netherlands (*P* values not reported).^51^ Similarly, a study in Germany reported statistically significant differences in Kiddy KINDL (4–6 years)/Kid‐KINDL (7–17 years) scores in DS patients versus age‐ and sex‐matched patients with drug‐resistant focal epilepsies (DRE, but no DEEs) and patients with seizure remission (SR) for total scores, emotional well‐being, friends and school; physical well‐being, and self‐esteem were significantly lower compared to SR patients (Table S3 and Figure 2).^54^ Patient EQ‐5D‐5L scores were evaluated in the DISCUSS study, a large on‐line survey of caregivers across Europe (N = 584 caregivers of pediatric [83%] and adult [17%] patients with DS), reporting scores of 0.42 in the whole cohort^46^; country‐specific values were 0.38 in the UK^48^ and 0.6 in Germany (vs 0.88 in the German general population).^56^ Results from the large European DISCUSS study reported no differences in EQ‐5D‐5L index values across age groups, that is, 2–5 years, 6–11 years, 12–17 years and ≥18 years,^46^ while studies in the UK,^44^ and the Netherlands^51^ reported that HRQoL assessed using the PedsQL was worse in older age groups. In the UK study, total and physical, psychosocial and cognitive function domain scores decreased with increasing age (from 2–3 years to ≥15 years),^44^ and similarly in the Dutch study, the physical functioning and social functioning scores decreased (worsened) with increasing age cohorts (1.5–5 years, 6–17 years and ≥18 years).^51^

The impact of seizures on HRQoL was analyzed in some studies,^43^, ^44^, ^46^, ^47^, ^55^ with fewer seizure‐free days,^43^, ^47^, ^55^ higher seizure frequency,^43^, ^46^, ^47^, ^55^ and a shorter interval without seizures^55^ having a negative impact on HRQoL. A study in the UK also found that young age at seizure onset, presence of myoclonic seizures and greater epilepsy severity were among the independent predictors of poor HRQoL.^44^ Comorbidities also adversely impacted HRQoL including behavioral difficulties,^44^, ^45^, ^51^ motor/walking difficulties,^44^, ^45^ and cognitive impairment/learning difficulties.^44^, ^51^

Mean caregiver EQ‐5D (3 L and 5 L) index scores (0 [worst]–1 [best]) ranged from 0.8 to 0.9 and VAS scores (0 [worst]–100 [best]) from 67 to 73 across three publications.^53^, ^54^, ^58^ In a study in Germany, scores were comparable in caregivers of patients with DS, DRE, and SR (age‐ and sex‐matched), however, a higher proportion of DS caregivers reported depressive symptoms assessed using the Beck Depression Inventory‐II (50% mild‐to‐severe) compared to caregivers of patients with DRE (25%) or SR (13%).^54^ Other studies also reported that a proportion of DS caregivers had symptoms of depression (70% slight and 34% moderate in the study by Campbell 2018 in the US,^58^ 47% in the study by Huang 2021 in Taiwan,^59^ and 66% in a global study by Villas 2017^65^). Common themes across the studies were that caring impacted on the personal/leisure time,^56^, ^57^, ^58^, ^60^, ^61^, ^62^, ^63^ energy,^61^, ^62^, ^63^ sleep,^50^, ^61^, ^62^ and social life of caregivers,^56^, ^60^, ^61^, ^62^ as well as on siblings and other family members,^56^, ^57^, ^60^ including effects on family vacations (i.e., not taking vacations or taking shorter vacations/vacations not far from home).^57^, ^60^, ^63^ Caregivers' concerns regarding the patient included the patient's future/lack of independence,^51^, ^56^, ^60^ seizure control,^56^, ^60^, ^66^ challenges regarding speech, communication, behavior and sleep,^50^, ^51^, ^56^, ^66^ and treatment‐related adverse effects,^55^, ^56^, ^60^ while there were also concerns about the impacts on siblings and other family members,^56^, ^60^ social isolation,^60^, ^66^ and having more children.^60^ Many parents (especially mothers^63^) slept with their child due to concerns about nocturnal seizures, the child's night waking and fear of SUDEP, leading to poor sleep quality.^50^, ^63^, ^64^, ^65^ An impact on finances was also commonly reported.^53^, ^54^, ^56^, ^57^, ^58^, ^60^, ^63^, ^67^ Across seven studies,^53^, ^54^, ^56^, ^57^, ^60^, ^63^, ^67^ caregivers (especially mothers^53^, ^63^) reported reducing their working hours (29%^53^, ^54^) and missing days from work due to caregiving (40%–65%),^53^, ^60^ while 28%–44% caregivers reported not working because of caregiving.^53^, ^54^, ^57^, ^60^, ^63^, ^67^ One study that compared the HRQoL of mothers and fathers found that mothers had a worse perception of their own general health.^63^ As with the patients, the HRQoL of caregivers was impacted by the severity of the patient symptoms.^47^, ^57^

### 3.4 Direct and indirect costs and healthcare resource utilization

Eleven studies published from 2014 to 2022 reported on the direct costs of DS, including five from Germany,^39^, ^53^, ^54^, ^56^, ^68^ four from the US,^67^, ^69^, ^70^, ^71^ and one from the Czech Republic^72^ and one from the EU 5 (the DISCUSS study)^60^ (Table S4). As with the other outcomes, there was substantial heterogeneity across studies, including population, data sources and included cost components. The risk of bias in the studies reporting on costs and/or resources was assessed to be low in seven studies and moderate in four studies (Table S9).

**FIGURE 3:**
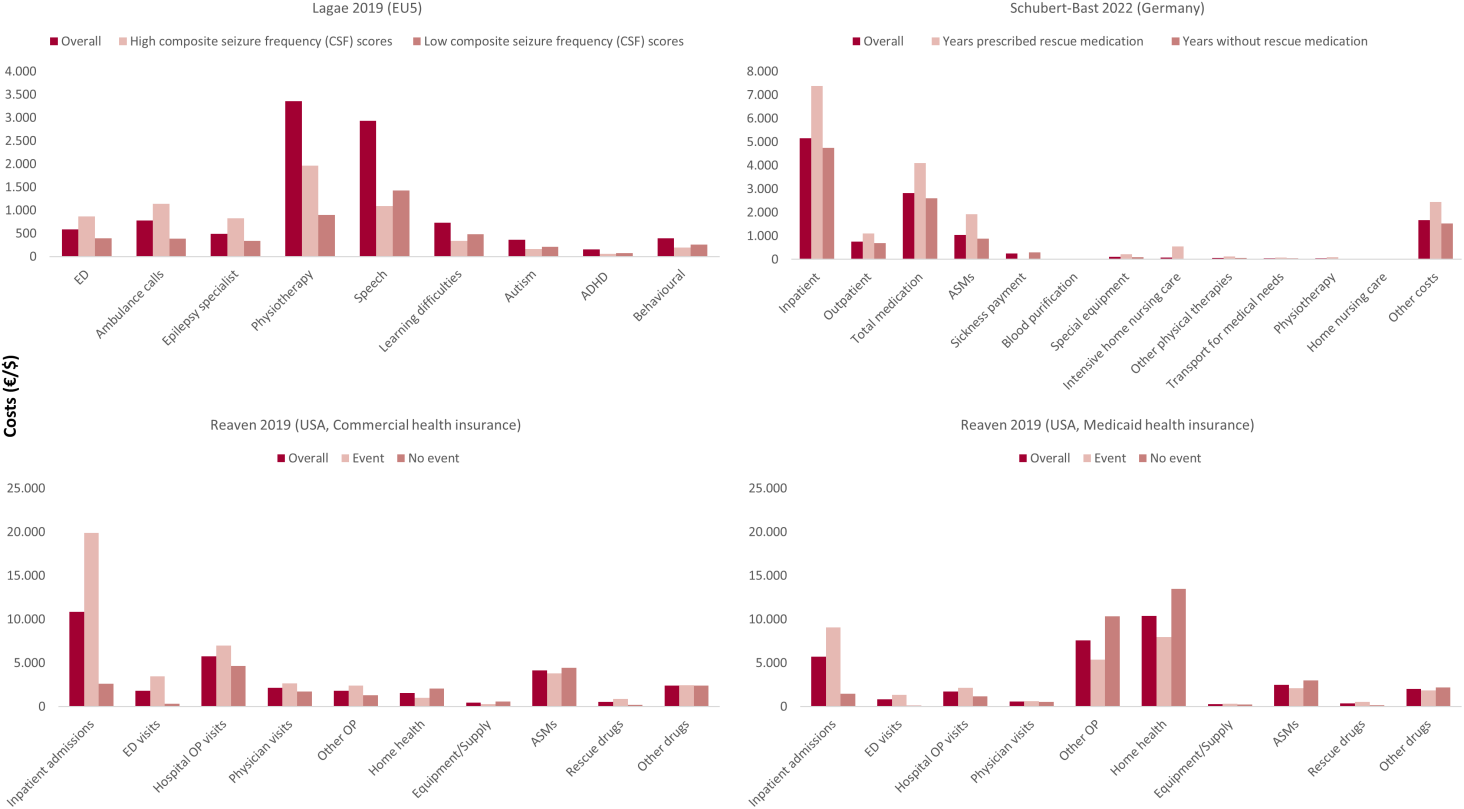
Direct costs in patients with DS according to seizure burden. ADHD, attention deficit hyperactivity disorder; ASM, anti‐seizure medication; ED, emergency department; EU5, France, Germany, Italy, Spain, and the UK; OP, outpatient. Data are mean values per person per year. $1.00 = €1 (Google Finance 30th August 2022) i.e. US dollars and Euros were equivalent at the time of writing the narrative synthesis. Adapted from Lagae,^60^ Schubert‐Bast^39^ and Reaven.^70^

Mean total direct costs were high across all studies, ranging from €11 048–€24 172 PPPY across the European studies,^39^, ^53^, ^54^, ^60^ and from $27 000–$77 914 PPPY across the studies in the US.^67^, ^69^, ^70^, ^71^ Where reported, inpatient admissions were a key cost driver across most studies (means of €5147–€6808 PPPY across studies in Europe^39^, ^53^, ^54^ and $5694–$11 565 across studies in the US^67^, ^70^), encompassing 18%–47% of the corresponding total direct costs. The mean costs of ASMs ranged from €1043–€6103 PPPY across the European studies^39^, ^53^, ^54^, ^60^ and $2488–$4130 PPPY in the US (commercial and Medicaid databases, respectively).^70^ Costs related to inpatient, outpatient and emergency department visits were generally higher in DS patients with seizure events (i.e., seizure events vs no events,^70^ high vs low composite seizure frequency [CSF] scores,^60^ and patients prescribed rescue medication vs not rescue medication^39^) (Figure 3).

Three publications reported indirect costs related to lost productivity^53^, ^54^, ^67^: one from the US ($19 925 PPPY) and two in Germany (mean [PPPY]: €19 160 and €19 028, including one study that reported lost productivity costs of €17 596 for mothers versus €1564 for fathers^53^). The study from the US also reported costs due to lost leisure time, equating to total indirect costs of $81 582.^67^ The DISCUSS study reported that out of pocket (OOP) expenses (i.e., co‐pay for therapies and healthcare) varied considerably across the European countries for epilepsy specialist fees, ASMs, and physical, speech, learning and behavioral impairments including autism and ADHD; median annual costs were estimated at between €1213–3303 per therapy.^60^ In Germany, mean OOP (health care costs, equipment expenditure, child care expenses for the DS child, child care expenses for siblings, travel expenses for appointments and home teaching expenses) were estimated at €4604 PPPY.^53^ One study in Japan reported on the difficulties that caregivers of patients with DS experienced in accessing the available medical expense subsidies.^73^

Fifteen publications, published from 2014 to 2022, reported on resource use including five from Germany,^39^, ^53^, ^54^, ^56^, ^68^ three from multiple European countries,^46^, ^60^, ^74^ three from the UK,^38^, ^48^, ^75^ four from the US,^69^, ^70^, ^71^, ^76^ one from Spain,^57^ and one from Taiwan^59^ (Table S5). Across the studies, the mean number of annual inpatient admissions ranged from 0.4 to 4.3,^38^, ^39^, ^53^, ^70^ the mean LOS in hospital per admission ranged from 0.6 to 25.6 days,^38^, ^39^, ^53^, ^54^, ^76^ and the mean number of ASMs ranged from 2.2 to 3.14.^37^, ^39^, ^46^, ^53^, ^54^, ^56^, ^68^, ^71^ Common ASMs were valproate (used by 63%–86% of patients across studies), clobazam (40–86%), stiripentol (STP) (26–91%) and topiramate (24%–77%)^38^, ^46^, ^53^, ^54^, ^56^, ^59^, ^74^; bromide was frequently used for patients in Germany (39%–49%),^53^, ^54^, ^56^ and levetiracetam was used in 55% of patients in Taiwan.^59^ Five studies reported that a proportion of patients were currently taking or had previously received ASMs that may exacerbate seizures in DS, including the sodium channel blockers lamotrigine and carbamazepine.^38^, ^46^, ^57^, ^59^, ^74^ Several studies reported that resource use was higher in those with a higher seizure burden,^39^, ^46^, ^54^, ^60^, ^70^, ^75^ including epilepsy specialist visits, emergency admissions, ambulance calls, inpatient admissions, hospital outpatient visits, physiotherapy visits, medication, services, and devices (especially intensive nursing care and special equipment), as well as annual hospitalization rate and LOS.^39^, ^60^, ^70^, ^75^ Increases in resources resulted in higher costs as discussed above (Figure 3).

## 4 DISCUSSION

**FIGURE 4:**
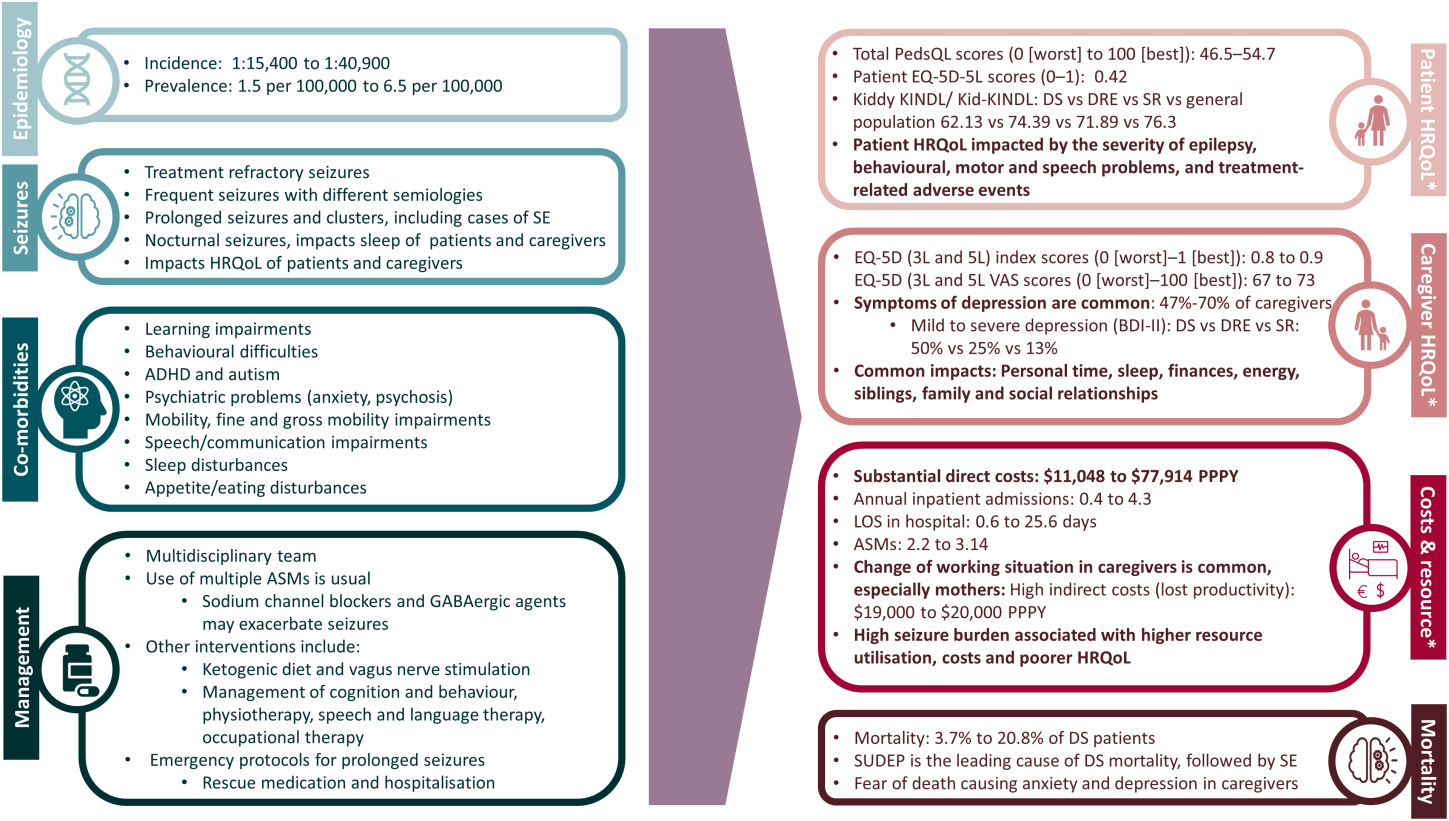
Summary of the burden of illness in patients with DS. ADHD, attention deficit hyperactivity disorder; ASM, anti‐seizure medication; BDI, Beck's Depression Inventory; DRE, drug‐resistant epilepsy; DS, Dravet syndrome; HRQoL, health‐related quality of life; LOS, length of stay; PPPY, per person per year; SE, status epilepticus; SR, seizure remission; SUDEP, sudden unexpected death in epilepsy; VAS, visual analogue scale. *Data are mean values across studies; Euros (€) were converted to US dollars at a rate of €1 = $1.00 (Google Finance 30th August 2022) i.e. US dollars and Euros were equivalent at the time of writing the narrative synthesis.

Overall, the burden of DS on patients, caregivers, the healthcare system and society is profound (Figure 4). The findings of this comprehensive SLR establish that DS is a rare disease, associated with impaired HRQoL for patients and caregivers (particularly mothers), and a substantial economic burden. The findings reflect the severe nature of DS, characterized by periods of distressingly frequent seizures and multiple neurodevelopmental impairments that require a multidisciplinary healthcare team, multiple ASMs and other interventions (e.g., physiotherapy) to manage.^6^ The severity of DS also impacts family life, particularly the lives of mothers; the studies describe how caregivers have little time for themselves, their sleep can be disrupted and there is often an impact on their jobs (e.g., having to take time off work/reduce hours or stop work altogether to attend appointments and care for their child). Overall, DS is associated with a physical, emotional, and time impact on caregivers and symptoms of depression are common.

This SLR builds on the evidence from a previous SLR on the clinical, economic, and HRQoL burden (conducted in December 2020 by Sullivan et al),^12^ and SLRs specifically on caregivers' HRQoL (one conducted in March 2021^22^ and one conducted in May 2020^21^), which have also documented the substantial burden posed by DS. Although there was some overlap between our SLR and the SLR by Sullivan et al, there were also some differences in the outcomes; the SLR by Sullivan included studies reporting on the natural history and symptoms of DS (94 out of 103 identified studies), but that SLR did not report on resource use, prevalence/incidence and it included fewer studies reporting on patient and caregiver HRQoL. Overall, our SLR adds updated and new evidence, with different outcomes and discussion points, compared to previous SLRs.

The values of many of the outcomes varied widely both between studies and also within studies (as shown by the large SDs and ranges). With regard to costs, resource use and HRQoL, this may reflect the complex and heterogenous nature of DS that can vary in severity and frequency of seizures and comorbidities and response to the available treatments; it is clear that seizure events affect all of these outcomes. Many of the studies also included a range of ages, including both children and adults, so differences may also reflect the natural evolution of DS from childhood to adolescence and adulthood, whereby awake seizures become less frequent but nocturnal seizures and the comorbidity burden increases.^12^ However, of note, there has been a lack of studies exploring the impact of age on costs and resource use in DS and other DEEs.^12^, ^77^, ^78^ Further differences in costs and resource use between the different studies may be due to differences in study design (e.g., data sources, included cost components, cost‐year, and different costs in different countries), as well as differences in access to different treatments and resources, which may be dependent on the funding of the healthcare systems (e.g., insurance based or government‐funded) and type of insurance.

The differences between studies for the epidemiology outcomes (incidence, prevalence and mortality) may be explained by differences in study design including population size, age, and definitions of DS. In general though, these outcomes may be underestimated as a diagnosis of DS may be initially missed by general healthcare professionals not trained to recognized the syndrome, and DS can be difficult to diagnose in adulthood. Studies have reported that some patients experience a considerable delay in diagnosis.^34^, ^41^, ^46^, ^49^, ^56^, ^57^, ^59^, ^72^ Early diagnosis in DS is particularly important because certain ASMs (e.g., sodium channel blockers or some GABAergic agents (e.g., gabapentin, pregabalin, tiagabine and vigabatrin) may exacerbate seizures, particularly in children,^6^, ^7^ and their long‐term use has been associated with poorer cognitive outcomes.^79^ Indeed, some studies in this SLR reported that a proportion of DS patients were taking or had previously taken these contraindicated ASMs.^38^, ^46^, ^59^, ^74^ In turn, seizure events lead to higher healthcare resource use (e.g., emergency treatment, including inpatient hospitalizations) and costs, as well as adversely impacting patient and caregiver HRQoL. In addition, while neurodevelopmental impairments may be caused by the underlying genetic defect in DS,^80^ a high seizure burden has been associated with having more comorbidities,^46^ and more severe physical and psychosocial functioning.^55^

The SLR was designed to look at the burden of DS in general without the impact of an intervention. However, of note, most of the included studies were conducted in patients prior to the approval of cannabidiol (CBD) (approved for the treatment of patients with DS in the USA in 2018 and in the EU in 2019) and fenfluramine (FFA) (approved in 2020 in the USA and EU) and studies did not include data on these ASMs (e.g., costs, proportion of patients using them). In addition, STP was only approved for patients with DS in the US in 2018, having been approved in the EU in 2007, and it was only used by a small proportion of patients in some studies. It will also be of interest to determine how these treatments impact the burden of illness in DS patients in general in future studies, especially as seizure events appear to be related to higher costs, resource use and lower HRQoL. In the randomized placebo‐controlled clinical trials, open‐label extensions and real‐world studies, CBD,^81^, ^82^, ^83^, ^84^, ^85^, ^86^, ^87^, ^88^ FFA^89^, ^90^, ^91^, ^92^, ^93^ and STP^94^, ^95^, ^96^, ^97^, ^98^, ^99^, ^100^, ^101^ have been associated with reductions in convulsive seizures,^81^, ^82^, ^83^, ^84^, ^85^, ^86^, ^87^, ^89^, ^90^, ^91^, ^92^, ^93^, ^94^, ^95^, ^96^, ^97^, ^98^, ^99^, ^100^, ^101^ increases in seizure‐free days,^88^, ^91^, ^102^ and seizure‐free intervals,^89^, ^90^, ^102^ improvements in the Caregiver Global Impression of Change,^81^, ^82^, ^85^, ^86^, ^89^, ^90^, ^91^, ^92^, ^93^ and a generally good tolerability profile.^81^, ^82^, ^83^, ^84^, ^85^, ^86^, ^87^, ^89^, ^90^, ^91^, ^92^, ^93^, ^94^, ^95^, ^96^, ^97^ Unlike some ASMs, psychiatric and behavioral side effects occur infrequently.^103^ In addition, FFA has been associated with improvements in executive function in responders,^104^ although more evidence is needed on the impact of CBD, STP and FFA on DS‐associated comorbidities. Because a high seizure burden (i.e., seizure frequency, fewer seizure‐free days, and a short seizure interval), comorbidities and treatment‐related adverse events have been associated with poorer HRQoL (as described herein), it is expected that improvements in these outcomes will lead to better HRQoL for patients and caregivers. Indeed, caregivers (n = 65) of patients with DS treated with FFA have reported improvements in patient cognitive function, alertness, and academic performance, and in their own sleep quality and feelings of stress and being overwhelmed,^105^ although further studies are needed to confirm these results. In addition, FFA may be associated with reductions in episodes of SE, SUDEP, and all‐cause mortality,^91^, ^106^ while STP has also been associated with reductions in prolonged seizures, episodes of SE, use of emergency medication and frequency of emergency/hospital visits.^68^, ^94^, ^95^, ^100^ Seizure events were also associated with higher healthcare resource utilization and direct costs. In this respect, initial evidence from health economic models suggests that CBD and FFA are cost‐effective treatments compared to usual care.^107^, ^108^ Of note, the cholesterol 24‐hydroxylase inhibitor soticlestat has shown reductions in convulsive seizure frequency in a Phase 2 trial,^109^ while therapies that aim to correct the underlying genetic defect in DS, including the antisense oligonucleotide STK‐001 and the cell‐selective gene therapy ETX101 are both in Phase 1/2a clinical development.^110^, ^111^ The ketogenic diet has also been found to be effective in a proportion of patients with DS, although it can be challenging to maintain.^112^

Challenges in conducting HRQoL evaluations for chronic childhood diseases are well documented.^113^ A caregiver proxy is required to inform patient HRQoL due to the patient's young age, cognitive impairment and not knowing a time before the condition. To provide a more standardized/objective assessment, the studies used quantitative measures including well‐used and validated generic tools such as the PedQL and the Kiddy KINDL/Kid‐KINDL. However, it is still possible that caregivers' perceptions of their child's HRQoL may be influenced by their own HRQoL, circumstances and concerns, especially regarding emotional aspects. Recruitment of caregivers through patient advocacy groups may also have contributed to bias, although the biases may be bidirectional, for example, some caregivers may have joined patient advocacy groups because their child has severe symptoms and they lack support, while others may have joined because they are highly engaged, have lots of support and more time. The majority of the recruited caregivers were mothers, and only one study looked at the difference in HRQoL between mothers and fathers, with mothers reporting poorer general health compared to fathers.^63^ Only a few studies reported HRQoL according to different age groups,^44^, ^46^, ^51^ two of which suggested that HRQoL was poorer in older age groups, which may reflect the increased impact of comorbidities on the patient's daily life. With a lack of expertise in adult healthcare provision and many adult DS patients still being seen by pediatric neurologists, evidence also suggest that the transition from pediatric to adult care can be challenging for caregivers and patients,^6^, ^114^, ^115^, ^116^ although we did not identify any studies specifically evaluating how the transition of care impacts the HRQoL of patients and caregivers.

While several studies reported that caring for a child with DS affected employment, estimates of indirect costs were only reported in three publications; the two in Germany focused on lost productivity whereas the one from the US also included lost leisure time. In addition, studies related to OOP expenses were mainly from Europe with a large proportion of patients from countries with full reimbursement systems (National Health Services)^53^, ^60^; these expenses may therefore be higher in countries that require partial or full payment for therapies and healthcare services such as the US.

It is noteworthy that most publications identified in this SLR were from the US or Europe, and no studies conducted in low‐to‐middle income countries were identified. Indeed, many of the outcomes, especially mortality, costs and resources (including available ASMs), are likely to be vastly different to those from high‐income countries, with higher mortality amid constrained resources having been reported for epilepsy in general.^117^, ^118^, ^119^ More research and innovative solutions are needed to improve patient outcomes in such regions.^120^, ^121^, ^122^, ^123^

The SLR was performed according to the PRISMA guidelines with search terms developed by an experienced information scientist, and there were no limits on language or date (except for abstracts). However, by focusing the search on Dravet syndrome/Severe Myoclonic Epilepsy of Infancy (SMEI) we may have missed some publications that included DS patients under the umbrella of drug‐resistant epilepsy (and related terms), although we tried to mitigate this by hand‐searching reference lists of other reviews/SLRs. In addition, while we searched major databases of relevance to this SLR, we may have missed studies from other databases or from searching the gray literature.

## 5 CONCLUSIONS

Overall, this SLR provides a comprehensive overview of the substantial burden that DS poses on patients, caregivers, healthcare resource utilization and costs. A high seizure burden and comorbidities are associated with poorer HRQoL and higher resource utilization and costs. More studies on the burden of illness in DS patients in low and middle‐income countries are needed, as well as studies on indirect costs in all regions. Furthermore, future studies will be able to assess the impact that newly approved ASMs such as CBD and FFA have on the burden of DS.

## AUTHOR CONTRIBUTIONS

All authors were involved in the conception and design of the study, acquisition and analysis of data and drafting of the manuscript and/or figures.

## CONFLICT OF INTEREST STATEMENT

AS reports personal fees and grants from Angelini Pharma (Arvelle), Biocodex, Desitin Arzneimittel, Eisai, Jazz (GW) Pharmaceuticals, Marinus Pharma, Precisis, Takeda, UCB Pharma (Zogenix), and UNEEG medical. LL received grants, and is a consultant and/or speaker for Zogenix; LivaNova, UCB, Shire, Eisai, Novartis, Takeda/Ovid, Epihunter, Jazz Pharmaceuticals. JW receives an honorarium from Epillepsa for her work as an associate editor, she serves on the national (South African) advisory board for Sanofi. AB has received honoraria for presenting at educational events, advisory boards and consultancy work for Biocodex, Jazz Pharmaceuticals/GW Pharma, Encoded Therapeutics, Stoke Therapeutics and UCB/Zogenix. PS reports personal fees and grants from Angelini Pharma, Eisai, Jazz Pharmaceuticals Biomarin, UCB, Proveca, and Zogenix. FR reports personal fees from Angelini Pharma, Arvelle Therapeutics, Desitin Pharma, Eisai GmbH, GW Pharmaceuticals companies/Jazz Pharma, Roche Pharma and UCB, and grants from the Detlev‐Wrobel‐Fonds for Epilepsy Research, the German Research Foundation, the German Ministry of Education and Research, the LOEWE Programme of the State of Hesse, and the European Union. SSB reports personal fees from Eisai, Desitin Pharma, GW Pharmaceuticals companies, Ethypharm, UCB, and Zogenix. We confirm that we have read the Journal's position on issues involved in ethical publication and affirm that this report is consistent with those guidelines.

## Supporting information


Appendix S1

Appendix S2
