# Supplementary material for: Dravet syndrome: A systematic literature review of the illness burden
Source: Epilepsia Open. 2023 Oct 11;8(4):1256–70. doi: 10.1002/epi4.12832 (PMC10690674; doi:10.1002/epi4.12832)
Supplement: Supplementary file 1 — Appendix S1 [file EPI4-8-1256-s001.docx]

**Dravet syndrome: a systematic literature review of the illness burden**

**Data extraction variables**

The following pre-specified variables were data extracted. Data were extracted for patients with DS, DS subpopulations (e.g patients with and without seizures and with and without rescue medication) and control populations used by the authors (e.g age matched non-DS patients).

- Study characteristics
  - Study type, country, observation period, population definition and number, age (mean (SD)/median (range)), percentage of male patients
- Epidemiology
  - Incidence, prevalence and mortality as reported by the authors
- Patient and caregiver HRQoL
  - Quantitative HRQoL measures for patients or caregivers (measure used, patient or caregiver and value) as reported by the authors
  - Measures included, but were not limited to, PedsQL, EQ-5D-5L/3L, Kiddy KINDL/ Kid-KINDL, quality of sleep, Beck's Depression Inventory (BDI), Oberst Caregiving Burden Scale (OCBS)
  - Impact on caregiver employment (proportion (%) of caregivers who quit work, reduced hours, and missed days)
  - Qualitative HRQoL studies were described descriptively
- Direct costs
  - Study type and source of data, country, cost year and inclusion year
  - Mean (SD) total direct costs and a breakdown of costs for individual healthcare resources as reported by the authors
  - Key conclusions
- Indirect costs
  - Study type and source of data, country, cost year and inclusion year
  - Mean (SD) total indirect costs, lost productivity costs, out of pocket (OOP) expenses
  - Key conclusions
- Healthcare resource utilization
  - Mean and standard deviation [SD] per person per year [PPPY] as reported by the authors including inpatient, outpatient, home nursing, equipment, physiotherapy, medications
  - Proportion (%) of patients using resources (e.g emergency department, ambulances)
  - Annual hospitalisation rates
  - Length of stay (LOS) in hospital and ICU
  - Anti-seizure medication use: mean (SD) number, proportion of patients taking 2, 3 or 4 ASMs and most commonly used (%)

**Search terms**

Conceptual Purpose: The aim of this search is to identify evidence for prevalence/incidence, healthcare resource utilization, costs, HRQoL (patient and caregiver) and mortality data for individuals with Dravet syndrome.

| Database | N |
| --- | --- |
| MEDLINE (MEDALL) | 578 |
| Embase | 735 |
| PsycINFO | 101 |
| Cochrane CDSR | 5 |
| Epistemonikos | 48 |
| Total | 1467 |

Database: MEDLINE (MEDALL)

Host: Ovid

Data parameters: 1946 to June 10, 2022

Date of search: 13 June 2022

| **Search strategy** | **Search narrative** |
| --- | --- |
| 1     Epilepsies, Myoclonic/ (3364)  2     (Dravet* adj2 syndrome*).ti,ab,kw,kf,ot. (1321)  3     (severe myoclonic epilep* or SMEI).ti,ab,kw,kf,ot. (404)  4     1 or 2 or 3 (4054) | Lines 1-3 sets out the condition search terms.  Line 1 is the relevant controlled indexing term in MEDLINE.  Lines 2-3 focus on free-text terms which align with the controlled indexing term at line 1. |
| 5     Epidemiologic Studies/ (9109)  6     incidence/ or prevalence/ (597641)  7     (epidemiolo$ or prevalenc$ or incidenc$).ti,ab,kw,kf. (1873042)  8     5 or 6 or 7 (2033536) | This cluster of terms represents a search for prevalence or incidence. The cluster is based on the search filter developed by Royle and Waugh for epidemiology studies (1). |
| 9     exp economics/ (645682)  10     exp "Costs and Cost Analysis"/ (258406)  11     exp Economics, Hospital/ or Financial management, hospital/ (32856)  12     Economics, Medical/ (9201)  13     economics, nursing/ (4013)  14     economics, pharmaceutical/ (3065)  15     (economic* or cost or costs or costly or costing or expense or expenses or price or prices or pricing or  pharmacoeconomic* or expense or expenses or CEA or CUA or CBA or CMA).ti,ab,kw,kf. (1041930)  16     (resource*1 and (allocation or utili* or using or usage or use*1)).ti,ab,kw,kf. (281578)  17     (expenditure* not energy).ti,ab,kw,kf. (34847)  18     (value adj1 (money or monetary)).ti,ab,kw,kf. (784)  19     (budget* or fiscal or funding or financial or finance*).ti,ab,kw,kf. (213858)  20     (decision* adj2 (tree* or analy* or model*)).ti,ab,kw,kf. (32234)  21     (markov or monte carlo).ti,ab,kw,kf. (77092)  22     ((statistical or simulation$) adj2 model$).ti,ab,kw,kf. (53817)  23     9 or 10 or 11 or 12 or 13 or 14 or 15 or 16 or 17 or 18 or 19 or 20 or 21 or 22 (1901787) | This cluster of terms represents the search for resource use. The search filter is based on the NHS CRD EED filter and it has been enhanced by comparison with the CADTH economic evaluations/costs/economic models filter (2, 3). |
| 24     ("Quality of Life in Childhood Epilepsy" or QOLCE).ti,ab,kw,kf. (96)  25     ("Quality of Life in Epilepsy for Adolescents" or "QOLIE-AD-48").ti,ab,kw,kf. (27)  26     ("The Impact of Childhood Illness Scale" or ICI).ti,ab,kw,kf. (12578)  27     ("The Hague Restrictions in Childhood Epilepsy Scale" or HARCES).ti,ab,kw,kf. (6)  28     ("Quality of Life in Epilepsy Inventory for Adolescents" or "QoLIE-AD-48").ti,ab,kw,kf. (27)  29     ("Quality of Life in Pediatric Epilepsy" or QoLPES).ti,ab,kw,kf. (9)  30     ("Quality of Life in Childhood Epilepsy" or (QoLCE or "G-QoLCE")).ti,ab,kw,kf. (96)  31     ("Impact of Pediatric Epilepsy Scale" or IPES).ti,ab,kw,kf. (91)  32     ("Health-Related Quality of Life Measure for Children with Epilepsy" or "CHEQoL-25").ti,ab,kw,kf. (17)  33     DISABKIDS.ti,ab,kw,kf. (105)  34     ("Epilepsy and Learning Disability Quality of Life" or ELDQoL).ti,ab,kw,kf. (8)  35     ("Glasgow Epilepsy Outcome Scale" or "GEOS-YP").ti,ab,kw,kf. (7)  36     "Impact of Epilepsy Schedule".ti,ab,kw,kf. (1)  37     (QOLIE or "QOLIE-31" or "QOLIE-10").ti,ab,kw,kf. (585)  38     ("Pediatric Quality of Life Inventory" or "PedsQL").ti,ab,kw,kf. (2548)  39     ("visual analog scale" or VAS).ti,ab,kw,kf. (73336)  40     ("Beck Depression Inventory" or BDI).ti,ab,kw,kf. (16695)  41     ("Hospital Anxiety and Depression Scale" or HADS).ti,ab,kw,kf. (12246)  42     ("Burden Scale for Family Caregivers" or BSFC).ti,ab,kw,kf. (106)  43     24 or 25 or 26 or 27 or 28 or 29 or 30 or 31 or 32 or 33 or 34 or 35 or 36 or 37 or 38 or 39 or 40 or 41 or 42  (116684) | This cluster of terms represents the search for disease burden and health-related quality of life. The filter has been conceived in two parts:  i) lines 24-42 represents condition specific instruments. These were developed from an informal scoping of intervention studies, reference to a recent review, and the clinical knowledge of the authors (4-10).  ii) lines 44-71 is based on Paisley and Booth’s HRQoL filter, which has been compared to CADTH’s filter for Health Utilities, and adapted through development by the information specialist undertaking the searches (3, 11). |
| 44     (15D or 15-D or 15 dimension).ti,ab,kw,kf,ot,hw. (5855)  45     (eq-5d or eq5d or eq-5 or eq5 or EQ-5D-Y or euro qual or euroqual or euro qual5d or euroqual5d or euro qol or  euroqol or euro qol5d or euroqol5d or euro quol or euroquol or euro quol5d or euroquol5d or eur qol or eurqol or eur  qol5d or eur qol5d or eur?qul or eur?qul5d or euro$ quality of life or european qol or EQ-5D-3L).ti,ab,ot,hw,kw. (15079)  46     (sf6 or sf 6 or SF-6D or short form 6 or short-form 6 or short-form six or shortform 6 or sf six or sfsix or  shortform six or short form six).ti,ab,ot,hw,kw. (3209)  47     (sf10 or sf 10 or short form 10 or short-form 10 or short-form ten or shortform 10 or sf ten or sften or  shortform ten or short form ten).ti,ab,ot,hw,kw. (152)  48     (sf12 or sf 12 or short form 12 or short-form 12 or short-form twelve or shortform 12 or sf twelve of sftwelve or  shortform twelve or short form twelve).ti,ab,ot,hw,kw. (7001)  49     (sf16 or sf 16 or short form 16 or short-form 16 or short-form sixteen or shortform 16 or sf sixteen or sfsixteen  or shortform sixteen or short form sixteen).ti,ab,ot,hw,kw. (37)  50     (sf20 or sf 20 or short form 20 or short-form 20 or short-form twenty or shortform 20 or sf twenty of sftwenty or  shortform twenty of short form twenty).ti,ab,ot,hw,kw. (427)  51     (sf36 or sf 36 or short form 36 or short-form 36 or short-form thirty six or shortform 36 or sf thirtysix or sf  thirty six or shortform thirstysix or shortform thirty six or short form thirty six or short form thirtysix or short  form thirty six).ti,ab,ot,hw,kw. (28893)  52     (health utilities index$ or (hui or hui1 or hui2 or hui3 or hui4 or hui-4 or hui-1 or hui-2 or  hui-3)).ti,ab,ot,hw,kw. (2082)  53     ("time trade off" or "time tradeoff" or "time trade-off" or TTO).ti,ab,ot,hw,kw. (2145)  54     (standard gamble$ or SG).ti,ab,ot,hw,kw. (12890)  55     ("discrete choice" or DCE).ti,ab,ot,hw,kw. (8849)  56     (AQoL or "Assessment of Quality of Life").ti,ab,ot,hw,kw. (2164)  57     Quality-Adjusted Life Years/ (14837)  58     (HRQoL or HRQL or HQL or QoL or (quality adj3 life) or HYE or HYES or (health$ adj3 year$)).ti,ab,ot,hw,kw.  (444539)  59     quality of life/ (243565)  60     value of life/ (5787)  61     uncertainty/ (16062)  62     (uncertain$ or wellbeing or "well being" or rosser or "willingness to pay").tw. (322344)  63     (utilit$ or disutility$).ti,ab,kw,kf. (246192)  64     (illness state$1 or health state$ or health status or Quality adjusted life year$ or QALY or QALD or qale or  qtime or life year$ or ICER or "incremental cost").ti,ab,ot,hw,kw. (206347)  65     (burden and (disease or illness or caregiver or carer or carers or parent* or home)).tw. (122410)  66     (lost adj2 (productivity or work or employment or earnings)).ti,ab,kw,kf. (3243)  67     (((disability or diseas$) adj3 adjust$) or daly$).ti,ab,kw,kf. (11310)  68     (preference* adj3 (valu* or measur* or health or life or estimat* or elicit* or disease or score* or instrument  or instruments)).ti,ab,kf,kw. (13170)  69     (self report$ or (patient adj report$ adj outcome$)).ti,ab,kw,kf. (215517)  70     (mortality or death).ti,ab,kw,kf. (1566613)  71     ((survey or surveys) adj3 (caregiver or carer or carers or parent* or home)).ti,ab,kw,kf. (5513)  72     44 or 45 or 46 or 47 or 48 or 49 or 50 or 51 or 52 or 53 or 54 or 55 or 56 or 57 or 58 or 59 or 60 or 61 or 62 or  63 or 64 or 65 or 66 or 67 or 68 or 69 or 70 or 71 (2845024) |  |
| 73     8 or 23 or 43 or 72 (5925355)  74     4 and 73 (578) | Line 73 combines lines:   - 8 - epidemiological studies or study data; or - 23 - search terms for economic evaluations or costs data; or - 43 - condition specific QoL tools; or - 72 – a standard search filter for HRQoL and Patient Reported Outcome tools.   Line 74 combines the condition search terms (line 4) with the lines set out above, to complete the search of MEDLINE. |

Database: Embase

Host: Ovid

Data parameters: 1980 to 2022 Week 23

Date of search: 13 June 2022

| **#** | **Searches** | **Results** |
| --- | --- | --- |
| 1 | *severe myoclonic epilepsy in infancy/ | 1140 |
| 2 | (Dravet* adj2 syndrome*).ti,ab,kw,kf,ot. | 2190 |
| 3 | (severe myoclonic epilep* or SMEI).ti,ab,kw,kf,ot. | 618 |
| 4 | 1 or 2 or 3 | 2558 |
| 5 | incidence/ | 502611 |
| 6 | prevalence/ | 849647 |
| 7 | (epidemiolo$ or prevalenc$ or incidenc$).ti,ab,kw,kf. | 2543994 |
| 8 | 5 or 6 or 7 | 2813298 |
| 9 | exp economic evaluation/ | 331519 |
| 10 | health-economics/ | 30310 |
| 11 | exp health-care-cost/ | 316496 |
| 12 | exp pharmacoeconomics/ | 214109 |
| 13 | (economic* or cost or costs or costly or costing or expense or expenses or price or prices or pricing or pharmacoeconomic* or expense or expenses or CEA or CUA or CBA or CMA).ti,ab,kw,kf. | 1307425 |
| 14 | (resource*1 and (allocation or utili* or using or usage or use*1)).ti,ab,kw,kf. | 365877 |
| 15 | (expenditure* not energy).ti,ab,kw,kf. | 45989 |
| 16 | (value adj1 (money or monetary)).ti,ab,kw,kf. | 988 |
| 17 | (budget* or fiscal or funding or financial or finance*).ti,ab,kw,kf. | 294814 |
| 18 | (decision* adj2 (tree* or analy* or model*)).ti,ab,kw,kf. | 44044 |
| 19 | (markov or monte carlo).ti,ab,kw,kf. | 85758 |
| 20 | ((statistical or simulation$) adj2 model$).ti,ab,kw,kf. | 65389 |
| 21 | 9 or 10 or 11 or 12 or 13 or 14 or 15 or 16 or 17 or 18 or 19 or 20 | 2238813 |
| 22 | ("Quality of Life in Childhood Epilepsy" or QOLCE).ti,ab,kw,kf. | 150 |
| 23 | ("Quality of Life in Epilepsy for Adolescents" or "QOLIE-AD-48").ti,ab,kw,kf. | 48 |
| 24 | ("The Impact of Childhood Illness Scale" or ICI).ti,ab,kw,kf. | 16927 |
| 25 | ("The Hague Restrictions in Childhood Epilepsy Scale" or HARCES).ti,ab,kw,kf. | 11 |
| 26 | ("Quality of Life in Epilepsy Inventory for Adolescents" or "QoLIE-AD-48").ti,ab,kw,kf. | 48 |
| 27 | ("Quality of Life in Pediatric Epilepsy" or QoLPES).ti,ab,kw,kf. | 12 |
| 28 | ("Quality of Life in Childhood Epilepsy" or (QoLCE or "G-QoLCE")).ti,ab,kw,kf. | 150 |
| 29 | ("Impact of Pediatric Epilepsy Scale" or IPES).ti,ab,kw,kf. | 126 |
| 30 | ("Health-Related Quality of Life Measure for Children with Epilepsy" or "CHEQoL-25").ti,ab,kw,kf. | 26 |
| 31 | DISABKIDS.ti,ab,kw,kf. | 180 |
| 32 | ("Epilepsy and Learning Disability Quality of Life" or ELDQoL).ti,ab,kw,kf. | 13 |
| 33 | ("Glasgow Epilepsy Outcome Scale" or "GEOS-YP").ti,ab,kw,kf. | 9 |
| 34 | "Impact of Epilepsy Schedule".ti,ab,kw,kf. | 1 |
| 35 | (QOLIE or QOLIE-31 or QOLIE-10).ti,ab,kw,kf. | 1149 |
| 36 | ("Pediatric Quality of Life Inventory" or "PedsQL").ti,ab,kw,kf. | 4555 |
| 37 | ("visual analog scale" or VAS).ti,ab,kw,kf. | 111160 |
| 38 | ("Beck Depression Inventory" or BDI).ti,ab,kw,kf. | 26786 |
| 39 | ("Hospital Anxiety and Depression Scale" or HADS).ti,ab,kw,kf. | 21556 |
| 40 | ("Burden Scale for Family Caregivers" or BSFC).ti,ab,kw,kf. | 130 |
| 41 | 22 or 23 or 24 or 25 or 26 or 27 or 28 or 29 or 30 or 31 or 32 or 33 or 34 or 35 or 36 or 37 or 38 or 39 or 40 | 179403 |
| 42 | (15D or 15-D or 15 dimension).ti,ab,kw,kf,ot,hw. | 7319 |
| 43 | (eq-5d or eq5d or eq-5 or eq5 or EQ-5D-Y or euro qual or euroqual or euro qual5d or euroqual5d or euro qol or euroqol or euro qol5d or euroqol5d or euro quol or euroquol or euro quol5d or euroquol5d or eur qol or eurqol or eur qol5d or eur qol5d or eur?qul or eur?qul5d or euro$ quality of life or european qol or EQ-5D-3L).ti,ab,ot,hw,kw. | 29211 |
| 44 | (sf6 or sf 6 or SF-6D or short form 6 or short-form 6 or short-form six or shortform 6 or sf six or sfsix or shortform six or short form six).ti,ab,ot,hw,kw. | 4109 |
| 45 | (sf10 or sf 10 or short form 10 or short-form 10 or short-form ten or shortform 10 or sf ten or sften or shortform ten or short form ten).ti,ab,ot,hw,kw. | 236 |
| 46 | (sf12 or sf 12 or short form 12 or short-form 12 or short-form twelve or shortform 12 or sf twelve of sftwelve or shortform twelve or short form twelve).ti,ab,ot,hw,kw. | 13368 |
| 47 | (sf16 or sf 16 or short form 16 or short-form 16 or short-form sixteen or shortform 16 or sf sixteen or sfsixteen or shortform sixteen or short form sixteen).ti,ab,ot,hw,kw. | 67 |
| 48 | (sf20 or sf 20 or short form 20 or short-form 20 or short-form twenty or shortform 20 or sf twenty of sftwenty or shortform twenty of short form twenty).ti,ab,ot,hw,kw. | 542 |
| 49 | (sf36 or sf 36 or short form 36 or short-form 36 or short-form thirty six or shortform 36 or sf thirtysix or sf thirty six or shortform thirstysix or shortform thirty six or short form thirty six or short form thirtysix or short form thirty six).ti,ab,ot,hw,kw. | 55348 |
| 50 | (health utilities index$ or (hui or hui1 or hui2 or hui3 or hui4 or hui-4 or hui-1 or hui-2 or hui-3)).ti,ab,ot,hw,kw. | 3918 |
| 51 | ("time trade off" or "time tradeoff" or "time trade-off" or TTO).ti,ab,ot,hw,kw. | 3190 |
| 52 | (standard gamble$ or SG).ti,ab,ot,hw,kw. | 19036 |
| 53 | ("discrete choice" or DCE).ti,ab,ot,hw,kw. | 12848 |
| 54 | (AQoL or "Assessment of Quality of Life").ti,ab,ot,hw,kw. | 3531 |
| 55 | Quality-Adjusted Life Years/ | 31543 |
| 56 | (HRQoL or HRQL or HQL or QoL or (quality adj3 life) or HYE or HYES or (health$ adj3 year$)).ti,ab,ot,hw,kw. | 762712 |
| 57 | "quality of life"/ | 556220 |
| 58 | socioeconomics/ | 147506 |
| 59 | uncertainty/ | 41010 |
| 60 | (uncertain$ or wellbeing or "well being" or rosser or "willingness to pay").tw. | 406390 |
| 61 | (utilit$ or disutility$).ti,ab,kw,kf. | 339159 |
| 62 | (illness state$1 or health state$ or health status or Quality adjusted life year$ or QALY or QALD or qale or qtime or life year$ or ICER or "incremental cost").ti,ab,ot,hw,kw. | 233902 |
| 63 | (burden and (disease or illness or caregiver or carer or carers or parent* or home)).tw. | 198209 |
| 64 | (lost adj2 (productivity or work or employment or earnings)).ti,ab,kw,kf. | 4733 |
| 65 | (((disability or diseas$) adj3 adjust$) or daly$).ti,ab,kw,kf. | 15879 |
| 66 | (preference* adj3 (valu* or measur* or health or life or estimat* or elicit* or disease or score* or instrument or instruments)).ti,ab,kf,kw. | 17064 |
| 67 | (self report$ or (patient adj report$ adj outcome$)).ti,ab,kw,kf. | 289135 |
| 68 | (mortality or death).ti,ab,kw,kf. | 2202470 |
| 69 | ((survey or surveys) adj3 (caregiver or carer or carers or parent* or home)).ti,ab,kw,kf. | 7447 |
| 70 | 42 or 43 or 44 or 45 or 46 or 47 or 48 or 49 or 50 or 51 or 52 or 53 or 54 or 55 or 56 or 57 or 58 or 59 or 60 or 61 or 62 or 63 or 64 or 65 or 66 or 67 or 68 or 69 | 4118812 |
| 71 | 8 or 21 or 41 or 70 | 7849179 |
| 72 | 4 and 71 | 735 |

Database: PsycINFO

Host: Ovid

Data parameters: 1806 to June Week 1 2022

Date of search: 13 June 2022

| **#** | **Searches** | **Results** |
| --- | --- | --- |
| 1 | (Dravet* adj2 syndrome*).ti,ab. | 321 |
| 2 | (severe myoclonic epilep* or SMEI).ti,ab. | 84 |
| 3 | 1 or 2 | 362 |
| 4 | (Epidemiolo$ or prevalenc$ or incidenc$).ti,ab. | 211553 |
| 5 | exp Economics/ | 85108 |
| 6 | health-economics/ | 1086 |
| 7 | exp health-care-cost/ | 24368 |
| 8 | exp pharmacoeconomics/ | 282 |
| 9 | (economic* or cost or costs or costly or costing or expense or expenses or price or prices or pricing or pharmacoeconomic* or expense or expenses or CEA or CUA or CBA or CMA).ti,ab. | 246772 |
| 10 | (resource*1 and (allocation or utili* or using or usage or use*1)).ti,ab. | 107714 |
| 11 | (expenditure* not energy).ti,ab. | 8926 |
| 12 | (value adj1 (money or monetary)).ti,ab. | 480 |
| 13 | (budget* or fiscal or funding or financial or finance*).ti,ab. | 88503 |
| 14 | (decision* adj2 (tree* or analy* or model*)).ti,ab. | 9782 |
| 15 | (markov or monte carlo).ti,ab. | 8261 |
| 16 | ((statistical or simulation$) adj2 model$).ti,ab. | 8423 |
| 17 | 5 or 6 or 7 or 8 or 9 or 10 or 11 or 12 or 13 or 14 or 15 or 16 | 453878 |
| 18 | ("Quality of Life in Childhood Epilepsy" or QOLCE).ti,ab. | 53 |
| 19 | ("Quality of Life in Epilepsy for Adolescents" or "QOLIE-AD-48").ti,ab. | 20 |
| 20 | ("The Impact of Childhood Illness Scale" or ICI).ti,ab. | 1213 |
| 21 | ("The Hague Restrictions in Childhood Epilepsy Scale" or HARCES).ti,ab. | 6 |
| 22 | ("Quality of Life in Epilepsy Inventory for Adolescents" or "QoLIE-AD-48").ti,ab. | 19 |
| 23 | ("Quality of Life in Pediatric Epilepsy" or QoLPES).ti,ab. | 10 |
| 24 | ("Quality of Life in Childhood Epilepsy" or (QoLCE or "G-QoLCE")).ti,ab. | 53 |
| 25 | ("Impact of Pediatric Epilepsy Scale" or IPES).ti,ab. | 32 |
| 26 | ("Health-Related Quality of Life Measure for Children with Epilepsy" or "CHEQoL-25").ti,ab. | 15 |
| 27 | DISABKIDS.ti,ab. | 46 |
| 28 | ("Epilepsy and Learning Disability Quality of Life" or ELDQoL).ti,ab. | 6 |
| 29 | ("Glasgow Epilepsy Outcome Scale" or "GEOS-YP").ti,ab. | 7 |
| 30 | "Impact of Epilepsy Schedule".ti,ab. | 0 |
| 31 | (QOLIE or "QOLIE-31" or "QOLIE-10").ti,ab. | 346 |
| 32 | ("Pediatric Quality of Life Inventory" or "PedsQL").ti,ab. | 735 |
| 33 | ("visual analog scale" or VAS).ti,ab. | 5321 |
| 34 | ("Beck Depression Inventory" or BDI).ti,ab. | 15529 |
| 35 | ("Hospital Anxiety and Depression Scale" or HADS).ti,ab. | 4873 |
| 36 | ("Burden Scale for Family Caregivers" or BSFC).ti,ab. | 22 |
| 37 | 18 or 19 or 20 or 21 or 22 or 23 or 24 or 25 or 26 or 27 or 28 or 29 or 30 or 31 or 32 or 33 or 34 or 35 or 36 | 27588 |
| 38 | (15D or 15-D or 15 dimension).ti,ab. | 284 |
| 39 | (eq-5d or eq5d or eq-5 or eq5 or EQ-5D-Y or euro qual or euroqual or euro qual5d or euroqual5d or euro qol or euroqol or euro qol5d or euroqol5d or euro quol or euroquol or euro quol5d or euroquol5d or eur qol or eurqol or eur qol5d or eur qol5d or eur?qul or eur?qul5d or euro$ quality of life or european qol or EQ-5D-3L).ti,ab. | 2797 |
| 40 | (sf6 or sf 6 or SF-6D or short form 6 or short-form 6 or short-form six or shortform 6 or sf six or sfsix or shortform six or short form six).ti,ab. | 356 |
| 41 | (sf10 or sf 10 or short form 10 or short-form 10 or short-form ten or shortform 10 or sf ten or sften or shortform ten or short form ten).ti,ab. | 20 |
| 42 | (sf12 or sf 12 or short form 12 or short-form 12 or short-form twelve or shortform 12 or sf twelve of sftwelve or shortform twelve or short form twelve).ti,ab. | 1605 |
| 43 | (sf16 or sf 16 or short form 16 or short-form 16 or short-form sixteen or shortform 16 or sf sixteen or sfsixteen or shortform sixteen or short form sixteen).ti,ab. | 5 |
| 44 | (sf20 or sf 20 or short form 20 or short-form 20 or short-form twenty or shortform 20 or sf twenty of sftwenty or shortform twenty of short form twenty).ti,ab. | 63 |
| 45 | (sf36 or sf 36 or short form 36 or short-form 36 or short-form thirty six or shortform 36 or sf thirtysix or sf thirty six or shortform thirstysix or shortform thirty six or short form thirty six or short form thirtysix or short form thirty six).ti,ab. | 5714 |
| 46 | (health utilities index$ or (hui or hui1 or hui2 or hui3 or hui4 or hui-4 or hui-1 or hui-2 or hui-3)).ti,ab. | 711 |
| 47 | ("time trade off" or "time tradeoff" or "time trade-off" or TTO).ti,ab. | 446 |
| 48 | (standard gamble$ or SG).ti,ab. | 1106 |
| 49 | ("discrete choice" or DCE).ti,ab. | 1334 |
| 50 | (AQoL or "Assessment of Quality of Life").ti,ab. | 615 |
| 51 | (HRQoL or HRQL or HQL or QoL or (quality adj3 life) or HYE or HYES or (health$ adj3 year$)).ti,ab. | 85158 |
| 52 | "quality of life"/ | 44910 |
| 53 | uncertainty/ | 10695 |
| 54 | (uncertain$ or wellbeing or "well being" or rosser or "willingness to pay").tw. | 166193 |
| 55 | (utilit$ or disutility$).ti,ab. | 65965 |
| 56 | (illness state$1 or health state$ or health status or Quality adjusted life year$ or QALY or QALD or qale or qtime or life year$ or ICER or "incremental cost").ti,ab. | 23875 |
| 57 | (burden and (disease or illness or caregiver or carer or carers or parent* or home)).tw. | 23511 |
| 58 | (lost adj2 (productivity or work or employment or earnings)).ti,ab. | 880 |
| 59 | (((disability or diseas$) adj3 adjust$) or daly$).ti,ab. | 2117 |
| 60 | (preference* adj3 (valu* or measur* or health or life or estimat* or elicit* or disease or score* or instrument or instruments)).ti,ab. | 8540 |
| 61 | (self report$ or (patient adj report$ adj outcome$)).ti,ab. | 141497 |
| 62 | (mortality or death).ti,ab. | 122127 |
| 63 | ((survey or surveys) adj3 (caregiver or carer or carers or parent* or home)).ti,ab. | 3482 |
| 64 | 38 or 39 or 40 or 41 or 42 or 43 or 44 or 45 or 46 or 47 or 48 or 49 or 50 or 51 or 52 or 53 or 54 or 55 or 56 or 57 or 58 or 59 or 60 or 61 or 62 or 63 | 577783 |
| 65 | 4 or 17 or 37 or 64 | 1116497 |
| 66 | 3 and 65 | 101 |

Database: Cochrane database of systematic reviews

Host: Wiley interface

Data parameters: Issue 6 of 12, June 2022

Date of search: 13 June 2022

ID Search Hits

#1 MeSH descriptor: [Epilepsies, Myoclonic] this term only 67

#2 (Dravet* NEAR/2 syndrome*):ti,ab,kw 141

#3 (severe myoclonic epilep* or SMEI):ti,ab,kw 127

#4 #1 or #2 or #3 201

Database: Epistemonikos

Host: <https://www.epistemonikos.org/en/>

Date of search: 13 June 2022

("Dravet syndrome" OR "severe myoclonic epilepsy")
