# Supplementary material for: Dravet syndrome: A systematic literature review of the illness burden
Source: Epilepsia Open. 2023 Oct 11;8(4):1256–70. doi: 10.1002/epi4.12832 (PMC10690674; doi:10.1002/epi4.12832)
Supplement: Supplementary file 2 — Appendix S2 [file EPI4-8-1256-s002.docx]

**Dravet syndrome: a systematic literature review of the illness burden**

**Supplemental Table S1: Overview of the studies**

| **Study** | **Country** | **Study design** | **Outcome** | | | | | | |
| --- | --- | --- | --- | --- | --- | --- | --- | --- | --- |
|  |  |  | **Incidence/**  **prevalence** | **Mortality** | **Patient HRQoL** | **Caregiver HRQoL** | **Direct costs** | **Indirect costs** | **Resource utilisation** |
| Aledo-Serrano 2020 [1] Full | Spain | DISCUSS: EU-wide caregiver survey (Spanish results); N=57 families |  |  |  | • |  |  | • |
| Aras 2015 [2] Full | Europe | Caregiver web-based survey: N=274 |  |  |  |  |  |  | • |
| Auvin 2021 [3] Full | UK & France | Caregiver on-line surveys: hypothetical vignettes: N=98 (UK=58 & France =40) |  |  | • |  |  |  |  |
| Bayat 2015 [4]; Full | Denmark | Retrospective: N=17 | • |  |  |  |  |  |  |
| Bjurulf 2022 [5] Full | Sweden | Retrospective: N=55 | • | • |  |  |  |  |  |
| Brunklaus 2011 [6] Full | UK | Caregiver survey: N=163 |  |  | • |  |  |  |  |
| Brunklaus 2012 [7]  Full | UK | Retrospective: N=241 | • | • |  |  |  |  |  |
| Brunklaus 2019 [8];  Abstract | UK, Ireland & Australia | Long-term, perspective outcome study: N=103 |  | • |  |  |  |  |  |
| Campbell 2018 [9]  Full | USA | Caregiver survey: N=34 |  |  |  | • |  |  |  |
| Ceska 2021 [10]  Full | Czech Republic | Retrospective: N=12 |  |  |  |  | • |  |  |
| Cooper 2016 [11] Full | Australia (+ Canada, Israel, New Zealand, US, UK) | Retrospective: N=100 patients |  | • |  |  |  |  |  |
| deLange 2019 [12]  Full | Netherlands | Telephone interviews of caregivers or adult patients with DS: N=116 |  |  | • |  |  |  |  |
| Gara-Adams 2021 [13] Abstract | England, UK | Clinician interviews: N=18 |  |  |  |  |  |  | • |
| Genton 2011 [14]  Full | France | Retrospective: N=24 |  | • |  |  |  |  |  |
| Hollenack 2019 [15]  Abstract | USA | Insurance claims analysis: *Commercial:* N=210 with DS/ 17,427,685 members  *Medicaid:* N=514 with DS/7,865,949 members | • |  |  |  |  |  |  |
| Hollenack 2019 [16]  Abstract | USA | Insurance claims analysis: N=504 |  |  |  |  | • |  |  |
| Huang 2021 [17]  Full | Taiwan | Caregiver on-line questionnaires: N=38 |  |  |  | • |  |  |  |
| Hurst 1990 [18]  Full | USA | Retrospective: N=1 with DS/39,270 population | • |  |  |  |  |  |  |
| Lagae 2018 [19]  Full | EU5 | DISCUSS: EU-wide caregiver survey (EU5 results); N=584 |  |  | • |  |  |  |  |
| Lagae 2019 [20]  Full | EU5 | DISCUSS: EU-wide caregiver survey (EU5 results); N=584 |  |  |  | • | • | •* | • |
| Lo 2021 [21] Full | UK and Sweden | Caregiver interviews: hypothetical vignettes: N=100 |  |  | • |  |  |  |  |
| Nabbout 2018 [22] | France | Caregiver interviews: N=11 |  |  |  | • |  |  |  |
| Nabbout 2019 [23]  Full | Australia, Italy, UK, USA | Caregiver interviews: N=20 |  |  |  | • |  |  |  |
| Nabbout 2020 [24]  Full | France | Caregiver on-line survey: N=87 |  |  |  | • |  |  |  |
| Nolan 2006 [25]  Full | Canada | Caregiver interviews: N=24 |  |  |  | • |  |  |  |
| Owen-Pickrell 2022 [26] Full | UK | Retrospective analysis of electronic health records: N=54 | • |  |  |  |  |  | • |
| Pagano 2019 [27]  Abstract | UK | DISCUSS: EU-wide caregiver survey (UK results); N=72 |  |  | • |  |  |  | • |
| Paprocka 2021 [28] Full | Poland | Caregiver survey: N=55 |  |  | • |  |  |  |  |
| Reaven 2019 [29]  Full | USA | Insurance claims analysis: Commercial: N=321; Medicaid: N=668 |  |  |  |  | • |  | • |
| Rosander 2015 [30];  Full | Sweden | Retrospective: N=42 | • |  |  |  |  |  |  |
| Sakauchi 2011 a & b [31, 32] Full | Japan | Retrospective survey data: N=438 |  | • |  |  |  |  |  |
| Schoonjans 2019 [33] Full | Belgium | Caregiver questionnaire: N=56 |  |  | • | • |  |  |  |
| Schubert-Bast 2022 [34] Full | Germany | Insurance claims analysis: N=160 | • | • |  |  | • |  | • |
| Sinoo 2019 [35]  Full | Netherlands | Caregivers completed |  |  | • | • |  |  |  |
| Skluzacek 2011 [36]  Full | 46 countries | Retrospective survey data: N=833 |  | • |  |  |  |  |  |
| Steckler 2020 [37]  Abstract | UK | Caregiver questionnaire: N=70 |  |  |  | • |  |  |  |
| Stockl 2019 [38]  Abstract | USA | Insurance claims analysis: N=183 |  |  |  |  | • |  | • |
| Stockl 2019 [39]  Abstract | USA | Insurance claims analysis: Commercial N=174; Medicaid N=303 |  |  |  |  |  |  | • |
| Strzelczyk 2014 [40] Full | Germany |  |  |  |  |  | • |  | • |
| Strzelczyk 2019 [41] Full | Germany | Caregiver survey: N=93 |  |  | • | • | • | • | • |
| Strzelczyk 2019 [42] Full | Germany | Caregiver survey: N=93 |  |  | • | • | • | • | • |
| Strzelczyk 2022 [43] Full | Germany | DISCUSS: EU-wide caregiver survey (German results); N=68 |  |  | • | • | • |  | • |
| Strzelczyk 2022 [44] Full | Germany | Regression analyses of pervious data |  |  | • |  |  |  |  |
| Symonds 2021 [45];  Abstract | Scotland | Prospective national cohort study: N=NR | • |  |  |  |  |  |  |
| Umeno 2019 [46]Full | Japan | Retrospective: N=13 |  |  |  |  |  |  | • |
| Villas 2017 [47]  Full | Global (USA, UK, Europe, Australia, Canada, S/Central America) | Caregiver survey: N=256 |  |  |  | • |  |  |  |
| Whittington 2018 [48] Full | USA | Caregiver survey: N=34 |  |  |  |  | • | • |  |
| Wu 2015 [49] Full | USA | Retrospective: N=8 | • |  |  |  |  |  |  |

*Out of pocket expenses

**Supplemental Table S2: Epidemiology: Incidence/prevalence and mortality in patients with DS**

| **Study** | **Country** | **Study type, source of data & sample size** | **Definition of DS** | **Year** | **Population (n); age/% male** | **Incidence/prevalence** | | **Mortality** |
| --- | --- | --- | --- | --- | --- | --- | --- | --- |
|  |  |  |  |  |  | **Parameter** | **Value** |  |
| Hurst 1990 [18]; USA  Full  USA | USA | Retrospective  Review of data from the NCPP in Texas (US) including 39,270 children born between 1956–1966 followed for 7 years | Diagnostic criteria for DS:   - Normal development before onset of seizure activity - Repeated prolonged (>1S30 min) febrile seizures - Onset after febrile seizures of mixed/myoclonus epilepsy - Developmental slowing with onset of seizure activity - Evolving EEG patterns | 1956–1966 with 7–year follow-up | Children ≤7 years with diagnostic criteria for DS (n=1/39,270)  NR | Incidence | 1: 40,000 | NR |
| Genton 2011 [14];  France  Full | France | Retrospective  Patients seen at the Saint-Paul-Hospital Henri Gastaut, Marseille, France | NR | First referred 1970 and 1992 & followed up beyond 20 yrs of age | N=24 adults with DS  Age 20–50 yrs; 58% male | NR | NR | 20.8% (5: SUDEP=3 pts; SE=1; unknown=1) |
| Skluzacek 2011 [36]  US, UK, France, Canada  Full | 46 countries | Retrospective  Survey data from 833 individuals with DS from the IDEA League membership database from 46 countries | NR | 2000–2010 | N=833 children and adults  N=802 patients alive: Mean age 7.7 yrs (range: <1–36 yrs); 51% male | NR | NR | 3.7% (31/833 SUDEP=19 pts; SE=10; other =2) |
| Sakauchi 2011 a & b [31, 32]  Japan  Full | Japan | Retrospective  Survey data from 147 of 246 hospitals that treat epilepsy pediatric patients in Japan | NR | Up to July 2009 | N=623 children from 91 hospitals  N=438 children from 26 hospitals that reported mortality rates  NR | NR | NR | 10.1% (63/623; or 14.4% [63/438]: SUDEP=31 pts; SE=21; drowning=6; other =1) |
| Brunklaus 2012 [7]; UK  Full | UK | Retrospective  Patients referred for *SCN1A* testing at the only center in the UK | Diagnostic criteria for DS:   - Seizure onset in infancy, mainly triggered by fever and often prolonged; later occurrence of various other seizure types (febrile and afebrile) including focal seizures; atypical absences; tonic–clonic seizures; - Normal cognitive and motor development prior to seizure onset with subsequent slowing including plateauing or regression of skills   *SCN1A* mutation | 2005 – 2010 | N=241 children and adults with *SCN1A+* DS  Age: 6 months–42 yrs; 56% male | Incidence: 5-year birth cohort from 2003 to  2007 (N=88 children) | 1:40,900 | 6% (5/88 children aged 3–7 years (SUDEP=3 pts; SE=2) |
| Bayat 2015 [4] Denmark  Full | Denmark | Retrospective  Children seen at the Danish Epilepsy Center, the only center specializing in epilepsy in Denmark | Children aged 4–9 years  Diagnostic criteria for DS:   - Seizure onset in infancy, mainly triggered by fever and often prolonged; later occurrence of other seizure types (febrile and afebrile) including focal seizures; myoclonic seizures; atypical absences; tonic–clonic seizures; - Normal motor and cognitive development prior to seizure onset with subsequent slowing including plateauing or regression of skills.   *SCN1A* mutation | 2004 – 2009 | N=17 children with *SCN1A+* DS  NR; 53% male | Incidence | 1:22,000 | NR |
| Rosander 2015 [30]; Sweden  Full | Sweden | Retrospective  DS cases from all neuropaediatricians at university and county hospitals & clinical genetic laboratories in Sweden | Diagnostic criteria for DS:   - ICD-10 code and the diagnostic criteria for DS according to the 1989 classification of epilepsies. | 2007–2011 | N=42 children with diagnostic criteria for DS  Mean age: 7.8 yrs; Median age: 7 yrs (range 1–17); 43% male | Incidence | 1:33,000 (95% CI 1:20,400–1:56,200) | NR |
|  |  |  |  |  |  | Prevalence in children <18 years on December 31, 2011 | 1:45,700  (95% CI 1:33,800–1:63,400)  *2.2/100,000 |  |
| Wu 2015 [49];  USA  Full | USA | Retrospective  All infants born at Kaiser Permanente Northern California (N=125,547 births) | Diagnostic criteria for DS  (subgroup with *SCN1A* mutation)  Patients with a diagnosis of seizure identified from EMR (ICD codes, ASM use) were reviewed & diagnosed with DS if they met 4 of 5 criteria:   - Normal or near-normal cognitive and motor development before seizure onset; - ≥2 febrile or afebrile seizures before 1 year of age; - Seizure semiology consisting of myoclonic, hemiclonic, or generalized tonic-clonic - seizures; - ≥2 seizures lasting longer - than 10 minutes; - Failure to respond to first-line antiepileptic drug therapy with continued seizures after 2 years of age | 2007–2010 | N=8 infants with diagnostic criteria for DS  NR | Incidence | 1:15,700 (95% CI: 1: 8000 – 1:31,000  births). | NR |
|  |  |  |  |  | N=6 infants with diagnostic criteria for DS+ *SCN1A+* | Incidence | 1: 20,900 (95%  CI: 1: 9600 – 1: 45,700). |  |
| Cooper 2016 [11]  Australia (+ Canada, Israel, New Zealand, US, UK)  Full | Australia (+ Canada, Israel, New Zealand, US, UK) | Retrospective  100 unrelated participants recruited to the Epilepsy Genetics Research Program from a cohort of 277  patients with the typical electroclinical phenotype of DS | Patients with the typical electroclinical phenotype of DS:   - Onset in the first year of life of convulsive seizures which were hemiclonic or generalized; myoclonic seizures; other seizure types which could include focal seizures, absence seizures, atonic seizures, tonic seizures; - Normal development in the first year of life with subsequent slowing including plateauing or regression; - Generalized spike-wave activity and either normal MRI or non-specific findings. | NR | N=100 patients with the typical electroclinical phenotype of DS    NR; 39% male | NR | NR | Mortality rate: 15.84 per 1000 person-years (98% CI 9.01–27.85).  SUDEP rate: 9.32 per 1000 person-years (98%  CI 4.46–19.45).  Mortality=17 pts SUDEP=10  SE=4,  drowning=2 asphyxia=1 |
| Brunklaus 2019 [8]; UK  Abstract | UK, Ireland & Australia | Long-term, perspective outcome study | SCN1A+ DS | 2009 (9-year period) | N=103 | NR | NR | 7% (7/103)  SUDEP=4 pts, SE=1, acute respiratory distress due to flu=1, unknown=1 |
| Hollenack 2019 [15]  US  Abstract | US | Retrospective insurance claims analysis: IBM® MarketScan® Commercial & Medicaid databases | ≥1 diagnosis code for refractory epilepsy  ≥1 diagnosis code for intellectual disability/developmental delay,  ≥2 AEDs or a diagnosis code for febrile seizures,  <91 cumulative days’ supply for AEDs that exacerbate DS,  No diagnosis codes for LGS or abnormal brain imaging | *Commercial:* 2016– 2017  *Medicaid:* 2016 | *Commercial:* N=210 with DS/ 17,427,685 members  *Medicaid:*  N=514 with DS/7,865,949 members | Prevalence | *Commercial:* 12 per million  *1.2/100,000  *Medicaid:*  65 per million  *6.5/100,000 | NR |
| Symonds 2021 [45];  Scotland  Abstract | Scotland | Prospective national cohort study based on referral via clinicians and review of EEG records. | Epilepsies presenting before 3 years of age  ILAE 2017 classification | NR | NR | Incidence | 6.5:100,000 (95% CI 3.2–10.0)  * 1:15,385 | NR |
| Owen-Pickrell 2022 [26];  UK  Full | UK | Retrospective analysis of EMR from healthcare databases: Primary care data (CPRD) linked to secondary care data (HES), and general population mortality data (ONS) (13.7 million de-identified EMRs of children & adults) | Confirmed DS: DS Read Codes (F25G.11 or F25G.00)  Probable DS: ICD-10/CRPD Read Code for epilepsy plus prescription for stiripentol or potassium bromide | 1987–2018 | Overall: N=54  Confirmed DS: N=32  Probable DS: N=22  Mean age 7.6 (SD: 10.9); median 3.0 (range: 0.0; 45.0); 50% male | Prevalence in 2017 | Overall: 1.5/100,000  Confirmed: 1.1/100,000 Probable: 0.6/100,000 | NR |
| Schubert-Bast 2022 [34]  Germany  Full | Germany | Retrospective insurance claims analysis: Vilua Healthcare research database, entries for >4 million people i.e ~5% of the German population | Probable DS:  ≥1 ICD-10 diagnosis of G40 (epilepsy)/G41 (SE) and ≥1 prescription of stiripentol, potassium bromide, or VPA+CLB+other ASMs (identified in the context of the epilepsy diagnosis)  Exclusion of any other  identified epilepsy syndrome, abnormal brain development, or prescription of Na+ channel blockers | 2007–2016 | N=160 children & adults with probable DS over 10-year period  N=64 identified in 2016  Mean age 33.2 (range: 3–82) yrs; 48% male | Prevalence in 2016 (N=64) | 4.7/100,000 | DS vs. matched controls: 11.88% (19 /160) vs. 1.19% (172 /14,540) p < 0.001 |
| Bjurulf 2022 [5]  Sweden  Full | Sweden | Retrospective, population based  DS cases in Sweden sought from heads of all paediatric and neuropediatric  Departments, epilepsy nurses and all paediatric neurologists and Members of the Dravet Syndrome Association Sweden; patients previously identified in Rosander 2015 were included | Diagnostic criteria for DS:  Normal EEG and no preexisting cerebral lesion in a normal  infant;  Normal development until the first seizure occurring before  one year of age;  Refractory clonic or tonic-clonic seizures affecting one or both sides simultaneously or alternatively;  Exclusion of any other  identified epilepsy syndrome including negative PCDH19 analysis in SCN1A negative participants | 2000–2018 | N=55 children with DS  NR | Prevalence: N=48 living children and resident in Sweden on December 31st, 2018 | 1:45,000 (95% CI 1/35,000–1/63,000)  *2.2/100,000 | 13% (7/53)  (SUDEP=3 pts; pneumonia =2; pneumonitis=1; acute anoxic brain injury=1) |
|  |  |  |  |  |  | Cumulative incidence | 1:33,000  (95% CI 1/24,000–1/  51,000) |  |

CI, confidence interval; DS, Dravet syndrome; EMR, electronic medical records; IDEA League, International Dravet syndrome Epilepsy Action League; NCPP, Neurological and Communicative Disorders and Stroke Collaborative Perinatal Project (NCPP); NR, not reported; pts, patients; SE, status epilepticus; SUDEP, sudden unexpected death in epilepsy; yrs, years

**Supplemental Table S3: HRQoL**

| **Study & country** | **Study design** | **Patient** | | | | **Caregiver** | |
| --- | --- | --- | --- | --- | --- | --- | --- |
|  |  | **PedsQL** | **EQ-5D-5L** | **Other e.g VAS, TTO, Kiddy KINDL/** **Kid-KINDL** | **Disease severity** | **Impact on caregivers & family** | **Employment** |
| Aledo-Serrano 2020 [1]  Spain  Full | Survey of caregivers  Spanish cohort from the pan european Dravet Syndrome Foundation survey (DISCUSS)  N=57 families; mean age: 9 years |  |  |  | Relationship between greater severity of the disease (cases with at least six visits to the emergency room) and family impacts, such as the loss of hours of school and leisure for siblings, having less than two hours of parental time off per week, not traveling on vacation and, to a lesser extent, difficulty in social and family relationships | 51% of caregivers reported having less than one hour of personal time per week, and an additional 28% had less than one hour per day for themselves (2-5 hours per week).  87% of siblings missed school at least once in the last month and 58% had missed leisure opportunities  Majority either do not go on vacation (33%) or go with limitations (not very far, not for long) | 32% of caregivers were unemployed (78% of those quit due to caregiving); 21% of cases work only part time, while 28% work full time.  9% of caregivers work as self-employed.  85% of caregivers have missed work in the past month to attend to their child's needs. In addition, 79% stated that this situation had influenced her professional career. |
| Auvin 2021 [3]  Full  UK and France | Observational study: On-line surveys  Caregivers score health state vignettes for a hypothetical patient with DS  N=98 (UK= 58 and France = 40) |  |  | Seizures/seizure-free days per month: Mean patient VAS score  UK  4/32: 0.20  0/30: 0.75  France:  4/32: 0.23  0/30: 0.92 | Health states with fewer seizure-free days resulted in lower utility scores (P < 0.001), and seizure-free days had a greater effect on  utility than seizure frequency (P < 0.001) |  |  |
| Brunklaus 2011 [6]  Full  UK | Observational study: Questionnaires for caregivers of DS patients referred to the SCN1A molecular genetic  diagnostic service based at the Royal Hospital for Sick Children, Glasgow between November  2005 and February 2010  N=163 children and adults  . | Mean (SD)  DS vs published UK norms:  Total score: 46.85 (19.99) vs 84.61 (11.19) Physical  functioning: 44.12 (28.85) vs 89.06 (12.27)  Psychosocial  health: 49.70 (17.58) vs 82.21 (12.67)  Emotional  functioning: 63.07 (20.85) vs 78.28 (15.54)  Social  functioning: 44.15 (24.11) vs 86.82 (15.42)  School  functioning: 40.63 (20.28) vs 81.52 (16.09)  p<0.001 for all |  |  | Young age at seizure onset (p = 0.019), presence of myoclonic seizures  (p = 0.029), motor disorder (p = 0.048), learning difficulties (p = 0.002), epilepsy severity (p < 0.001), and behavioural difficulties (p < 0.001) each independently predicted  poorer HRQoL. Behavioural problems such as hyperactivity/ inattention were the strongest predictors of poorer HRQoL. |  |  |
| Campbell 2018 [9]  Full  USA | Single centre (Children's Hospital Colorado)  Caregiver survey  from November 30, 2016 to  December 20, 2016  N=34  2 to 22 years (mean ± SD, 11.7 ± 5.8 years). |  |  |  |  | OCBS: moderate to severe time and difficulty limitations when performing routine caregiving tasks: providing transportation (93% of respondents), personal [patient with  DS] care (87%), additional household tasks (83%), communication  (80%), and symptom observation (77%).  The top five domains with regard to proportion of caregivers reporting moderate or greater difficulty  included: arranging for care (73%), communication (70%), coordinating  resources (67%), managing behavior problems (67%), and personal  [patient with DS] care (63%).  Mean (SD) (range)  EQ-5D-5L Index (0–1): 0.78 (0.17) (range: 0.31–1)  VAS (0–100): 67 (21) (range: 11–94)  EuroQoL 5D-5L domains with the greatest impact on caregivers were anxiety/depression (70% of respondents ≥ slight problems, 34% ≥ moderate) and discomfort/  pain (57% of respondents ≥ slight problems, 23% ≥ moderate).  Respondents who scored <65 were two- to fourfold more likely to report ≥moderate time spent and difficulty managing child behaviour problems and assisting with walking, suggesting that children with DS with high degrees of motor or neurodevelopmental problems have an especially high impact on caregiver health. | Quit work: 45% |
| deLange 2019 [12]  Full  Netherlands | Single centre (University Medical Center Utrecht), telephone interviews of caregivers or patients (if adult and mentally competent) with DS (N=116; mean age: 15 (range: 4–67 years)) and non-DS syndromes (N=48; 22 (4–67) years) | Mean (range)  DS vs non-DS  52.6 (13–86) vs 88.5 (63–99) |  |  | Walking disabilities and severe behavioural problems are strongly correlated to lower HRQoL scores. |  |  |
| Huang 2021 [17]  Full  Taiwan | On-line questionnaire of caregivers of patients with a diagnosis of DS (diagnosed and actively followed up by a pediatric neurologist in Taiwan.  N=38  Mean age (SD): 10.5 (2.6 [range: 1–28]) years |  |  |  |  | Depression: 47%  Moderate or greater difficulty in performing (OCBS): additional household tasks: 79%  Observing and reporting  symptoms: 77%  Seeking further medical plans: 76%.  Financial issues: 66% Medical or nursing treatments 66%  Medication use: 63% Patient care: 58%)  Mobility problems: 50%.  Top three concerns included:  lack of  independence: 61%  Seizure control: 58% Speech and communication challenges: 50%  Impacts on siblings: 50% |  |
| Lagae 2018 [19]  Europe  Full | The Dravet syndrome caregiver on-line survey (DISCUSS survey)  N=584 caregivers of paediatric (83%) and adult (17%) patients with DS  Mean age: 10.6 years |  | Patient (EQ-5D-5L)  Mean (SD) (range)  0.42 (0.29) (range < 0 – 1) |  | High seizure frequency was related to a lower HRQoL (EQ-5D-5L). |  |  |
| Lagae 2019 [20]  Full  EU5 (France, Germany, Italy, Spain, UK) | DISCUSS EU5 cohort  Online survey in 2016. Caregivers of patients with DS answered questions related to the previous 3 months  N=584 caregivers of paediatric (83%) and adult (17%) patients with DS |  |  |  |  | Difficulties:  Daily activities: 91%  Family relationships: 70%  Social life: 80%  <1 hour  to themselves: 77%  Concerns:  medical treatments are working: 71.2%  Side effects of treatment: 82.5  How others will react: 36.3%  How illness is affecting other family members: 46.4%  Patient’s future: 95.7%  Having more children: 21.9%  Emotional impact on other children in family: 50%  65% reported travelling on  Holiday (approx. 50% in age groups vs approx. 70% in older groups)  but with limitations (e.g not going on plane)  Siblings missed leisure opportunities in past 4 weeks: 46% | 80% of caregivers reported an influence on their career choices  Unemployed (81% due to caregiving): 34%  Missed days: 65% |
| Lo 2021  Full [21]  UK and Sweden | Caregivers score health state vignettes for a hypothetical patient with DS based on seizure frequency.  N=100 Interviews from March to April 2020 regarding DS vignettes  UK and Sweden |  |  | Mean (SD)  Seizures/seizure-free days per month: 0/>24 vs >25/≤18  Patient VAS score: 63.1 (22.0) vs 22.1 (17.6)  Patient time trade-off (TTO): 0.778 (0.229) vs 0.171 (0.533)  Caregiver VAS score: 68.4 (19.0) vs 26.4 (17.8)  Caregiver time trade-off (TTO): 0.881 (0.153) vs 0.510 (0.457) | Fewer seizures and additional seizure-free days are associated  with better patient and caregiver HRQoL |  |  |
| Nabbout 2018 [22]  France  Full | Semi-structured interviews of  caregivers  of children with DS (aged 2–18 years old)  N=11 |  |  |  |  | Impacts:  Emotional  -Fear/afraid: 64%  -Worry: 64%  -Uncertainty: 36%  -Annoyed/frustrated: 36%  Physical:  -Tiredness: 45%  Family:  -Other family members: 55%  -spouse: 45%  Daily activities  -constant care of child: 55%  -disrupted daily routine: 55%  Leisure: 45%  -No time: 27%  Social life: 36%  Sleep: 45% | Unable to work: 36% |
| Nabbout 2019 [23]  Australia, Italy, UK, USA  Full | Semi-structured interviews of  caregivers  of children with DS (aged 2–18 years old)  N=20 |  |  |  |  | Impact on  Work-life: 90%  Family: 90%  Leisure: 80%  Financial wellbeing: 80%  Sleep disturbance: 75%  Daily activities: 70%  Physical functioning: 70%  Social functioning: 60%  Emotional experience: 50% |  |
| Nabbout 2020 [24]  France  Full | On-line survey between October 2014 and January 2015 to caregivers of children and adolescents aged under 18 years  with DS (Dravet Syndrome Alliance (France) and the Reference Centre for Rare Epilepsies (France).  Median age: 7.57 (IQR: 5.01–10.00) years  N=87 |  |  |  |  | Perception of health:  Own general health poor or very poor: 19.5% of mothers vs. 15.3% of fathers  Social life:  Care of their child with DS had an important impact on their time and energy: 85.1% of mothers vs 49.4% of fathers  Do not go on vacation: 18.4% | Difficulties finding or keeping job: 50% of mothers vs ~10% of fathers  Mothers not working: 33%  Parents estimated that they spent an average of around 4 h per week for attending therapy appointments |
| Nolan 2006 [25]  Full  Canada | Single centre, semi-structured interviews of caregivers of patients with DS  N=24 |  |  |  |  | Concerns and stresses about:   - Diagnosis - Seizure control - Developmental, behavioral, and sleep issues - Negative effects in relationships with others - Social isolation for parents as a result of child’s decreased   cognitive level and increased behavioral problems   - Respite and relief care were hard to obtain |  |
| Pagano 2019 [27]  Abstract  UK | Survey of caregivers  UK cohort from the pan european DISCUSS study  N=72  caregivers of paediatric (78%) and adult (22%) patients with DS  Mean age: 11 (range: <1–34) years |  | Mean (SD) (range)  Patient EQ-5D-5L  0.38 (0.27) (range -0.17 – 0.88) |  |  |  | Unemployed: 33%  Full-time carers: 7%  Missed average 6 hrs per week due to caring (e.g hospital appts): 53% |
| Paprocka 2021 [28]  Poland  Full | Survey of caregivers of patients with DS by the  members of the Polish support group of the Association for People with Severe Refractory Epilepsy  DRAVET.PL  N= 55 caregivers of patients with DS (mean: 9 years range 2–26 years). | Mean (SD) total score :  Males (N=30): 46.5 (22)  Females (N=25): 52 (16)  Key domains affected:  Physical:  - taking a bath/shower or performing  household chores  -walking more than 100 m  -Running  Emotional:  - Anger  -Sleep  Social:  -Not able to do things other children their age can do |  |  | The frequency  of adverse effects was related to the number of drugs used, which had an impact on the  PedsQL score, especially physical and social functioning |  |  |
| Schoonjans 2019 [33]  Belgium  Full | Online questionnaire based on the ‘Sleep Behavior Questionnaire by Simonds & Parraga (SQ-SP)’ completed by DS parents and a control group (parents from children with epilepsy).  N=56 (75% ≤ 18 years)  Mean age: 13.7 years (range: 1–48) years |  |  | Mean (SD)  Quality of sleep (QoS) (0 to 10 [best]): DS vs control (epilepsy)  6.5 (2.09) vs 7.14 |  | Child’s sleep problems had a negative impact: 46%  Impacts: waking from sleep, sleepiness during the day, difficulties with concentration and memory and feelings of helplessness and frustration  Mean (SD)  Quality of sleep (QoS; 0 to 10 [best])  DS vs control: 5.9 (2.14) vs 5.98 |  |
| Sinoo 2019 [35]  Full  Netherlands | Caregivers of patients with DS known to the University Medical Center  Utrecht completed the PedsQL  N=85 with DS  Mean age: 13.74 (9.09) (range: 2–44 years) vs N=31 non-DS epilepsy patients | Mean (SD)  Total score: 54.7 (16.5) vs 93.5 (4.8) vs published general population 83.0 (14.79)  Physical  functioning: 45.7 (27.0) vs 99.6 (1.1)  Psychosocial  health: 60.1 (14.9) vs 90.1 (7.4)  Emotional  functioning: 70.5 (15.7) vs 85.6 (18.2)  Social  functioning: 54.7 (24.4) vs 95.0 (7.6)  School  functioning: 55.3 (22.3) vs 92.4 (10.7) |  |  | - Problems with attention, aggression, and withdrawn behaviour were related to social functioning impacts. - Somatic problems and anxiety/ depression were related to emotional functioning impacts. - Cognitive impairment and behavior problems were both independent predictors of poorer HRQoL; behavior problems were the strongest predictor. - Seizure frequency was only indirectly related to HRQoL, mediated by cognitive impairment. | Worried about:  Self-reliance/future: 48%  Physical health: 25%  (Cognitive) development: 25%  Psychological and behavioural problems: 25%  Seizures/epilepsy: 20%  Communication/social contacts: 16% |  |
| Steckler 2020 [37]  UK  Abstract | 10-year follow-up study from 2009 study  Caregivers completed a structured postal questionnaire  N=70 |  |  |  | Poorer developmental quotients correlated with early onset of initial developmental concerns (rS=0.31; p=0.037), later mobility problems (rS=0.30; p=0.015), higher levels of behaviour problems (rS=0.26; p=0.043) and worse PedsQL scores (rS=0.31; p=0.015). | Health and wellbeing negatively affected: 98% | Quit work: 90% |
| Strzelczyk 2019 [41]  Germany  Full | Carers of patients with DS recruited from throughout centres in Germany  and through the German DS patient advocacy  group completed surveys  (German DS study)  N=93  Mean age: 10.1 (7.1) years (median: 8.7, range: 15 months–33.7 years) |  |  | Mean (SD)  Patients aged 4–6 years (KiddyKINDL): 65.0 (11.1), (range 39.6–82.3) vs 81.9 for general population  Patients aged 7–17 (Kid-KINDL): 54.4 (14.2) range 27.1–80.2) vs 77.0 for the general population |  | Caregiver depression (BDI-II >13 points): 45%  -mild: 22%  -moderate: 15%  - severe: 9%  Caregiver (EQ-5D-3L)  EQ-5D-3L index: 0.9 (0.18) (range 0.3–1) vs 0.9 in German general population  EQ-VAS: 71.3 (18.0) range 19–100) vs 77.3 in German general population  Some problems with anxiety/ depression: 38.2% vs 4.3% in German general population | Maternal vs paternal  Quit work: 31% vs 1%  Reduced hours at work: 29% vs 6%  Missed days: 40% vs 27% |
| Strzelczyk 2019 [42] | Patients with DS and their caregivers recruited from throughout centres in Germany  and through the German DS patient advocacy  group  DS vs DRE vs SR  German DS study & the EpiPaed study (completed in 2011 by 489  caregivers of children with epilepsy)  DS vs DRE vs SR  N= 93 vs 93 vs 93  Pediatric DS vs DRE vs SR  Mean age: 8.1 vs 7.6 vs 8.3 years  Adult DS vs DRE vs SR  Mean age: 24.6 vs 23.9 vs 23.6 years |  |  | Kiddy‐KINDL/Kid‐KINDL  DS vs DRE vs SR vs general population  Mean  Total: 62.13 vs 74.39 vs 71.89 vs 76.3  Physical well‐being: 62.77 vs 72.27 vs 73.88 vs 76.5  Emotional well‐being: 75.13 vs 79.82 vs 78.03 vs 80.8  Self‐esteem: 62.17 vs 66.47 vs 70.62 vs 68.8  Family: 75.04 vs 80.63 vs 75.75 vs 77.7  Friends: 47.55 vs 70.91 vs 70.93 vs 78.0    School: 57.85 vs 81.21 vs 74.41 vs 76.0  The KINDL subscale scores were lower in DS patients for  the domains “friends” (P < 0.001), “school” (P < 0.001), and  “well‐being” (P < 0.05), compared with patients in DRE and  SR cohorts. |  | Caregiver depression (BDI-II >13 points)  -mild: 26% vs 14% vs 8%  -moderate: 16% vs 9% vs 4%  - severe: 8% vs 3% vs 1%  Mean overall score: 14.9 vs 9.4 vs 6.9 (p<0.001 for DS vs DRE and vs SR)  Caregiver (EQ-5D-3L) DS vs DRE vs SR vs general population  Mean (SD) EQ-5D-3L index:  0.90 (0.18) vs 0.94 (0.10) vs 0.96 (0.07) vs 0.9  DS vs SR p<0.01  Mean EQ-VAS: 73 vs 76 vs 80 vs 77  DS vs SR p<0.01  Some problems with anxiety/ depression: 38.2% vs 4.3% in German general population | Quit work: 28%  Reduced hours at work: 29% |
| Strzelczyk 2022 [44]  Germany  Full | Regression analyses to evaluate the association between 3 composite scores (physical, psychosocial and care requirements) based on previous Kiddy‐KINDL/Kid‐KINDL data [41, 42]) and three forms of seizure measures (seizure frequency, days with no seizures and longest interval without seizures)  N=75 paediatric patients |  |  |  | Seizures: Strong associations were found between each of the three composite symptom scores and each of the three seizure measures, with the regression coefficient on symptom score highly significant (p≤0.001) in all nine comparisons.  Other manifestations:  Kiddy KINDL (4–13 years old; n=37): Behavioural/ attention problems and status epilepticus were significant predictors of  HRQoL.  Kid KINDL (14–17 years old) n=20: speech and language problems |  |  |
| Strzelczyk 2022 [43]  Germany  Full | Carers of patients with DS completed surveys  (German results from DISCUSS study)  N=68  Mean age: 10 years (median: 9, range: 1-26). |  | Mean (SD)  Patient  EQ-5D-5L index value: 0.60 (0.26) vs 0.88 for German general population |  |  | Impacts:  Daily life: 94.1%,  Family relationships: 76.5%,  Social life: 70.6%  Concerns:  Child's future: 98.5%, Tolerability or effectiveness of drug therapies: 86.8%, 69.1% | Quit work: ~33% |
| Villas 2017 [47]  Global (USA, UK, Europe, Australia, Canada, S/Central America)  Full | Survey conducted by the Dravet Syndrome Foundation (DSF)  N=256  Age range: 9 months – 32 years |  |  |  |  | Concerns about emotional Impact on siblings: 74%  Depression: 66%  Family Therapy: 26%  Bereavement counselling: 8%  Concerns (n):  Speech/communication (cannot vocalize pain): 43  Sibling impacts: 42  Cognitive/developmental delay/regression: 39  Behavioural issues including violence and autistic traits: 34  Long-term care when parents are gone: 27  SUDEP or death: 21  Lack of independence/ constant care; 19  Anxiety/depression/isolation (parent): 19  Mobility: 17  Finances (parent): 15  Quality of life (family): 15  Medication side effects: 8 |  |

BDI, Beck's depression inventory; HRQoL, health-related quality of life; IQR, interquartile range; OCBS, Oberst Caregiving Burden Scale; SD, standard deviation; SUDEP, sudden unexpected death in epilepsy; TTO, time trade-off; VAS, visual analogue scale

PedsQL scores: 0 (worst) to 100 (best); EQ-5D-5L scores: 0 (worst) to 1 (best); Kiddy KINDL/ Kid-KINDL scores: 0 (worst) to 100 (best); EQ-5D (3L and 5L) index scores: 0 [worst] to1 (best]): EQ-5D (3L and 5L) VAS scores: 0 (worst) to 100 (best); VAS: 0 (worst) to 1 (best) or 0 (worst) to 100 (best)

**Supplemental Table S4: Direct and indirect costs**

| **Study** | **Country** | **Study type, source of data** | **Definition of DS** | **Year (inclusion year/cost year)** | **Population (n); age/% male** | **Direct costs** | **Total direct costs, mean PPPY** €/US$ | **Indirect costs** | **Key conclusions** |
| --- | --- | --- | --- | --- | --- | --- | --- | --- | --- |
| Ceska 2021[10]  Full | Czech Republic | Retrospective  Patients treated at epilepsy centre in Czech Republic | - Confirmed SCN1A gene mutation. | Inclusion year: 2018  Cost year: 2018 | N=12  Mean age: 10.75 (6.26) years (median: 8.5, range: 3–19 years)  Male: 33% | **Inpatient and outpatient costs, mean per month (SD)**  Before vs after diagnosis  €204.5 (167.1) vs €29.4 (26.1)  **Key direct costs, mean per month (SD) (%)**  Hospitalization: €102.10 (50.2%) vs €10.3 (35%)  -Neurological department: €43.3 (52) (21.2%) vs 4.5 (5.7) (15.3%)  Non-hospitalisation components: €101.70 (49.8%) vs €19.1 (64.8%)  -Genetic tests: 75.8 (145.2) (37%) vs 0  -Rehabilitation: 1.3 (3.2) (0.6%) vs 5.1 (8.7) (17.3%) | NR | NR | DS results in essential health care utilization and high financial burden **before**  **diagnosis** caused by repeated hospitalization and extensive diagnostics tests |
| Hollenack 2019 [16]  Abstract | USA | Retrospective insurance claims analysis: IBM® MarketScan® Medicaid databases | Probable DS (or LGS or DRE)   - ≥1 ASM claim and medical claims with ≥1 diagnosis code for LGS or refractory epilepsy or ≥1 claim for clobazam or rufinamide. | Inclusion year: 2014–2015  Cost year: NR | *Medicaid:* Probable DS (N=504)  Mean age: 17.7 years  Male: 54.8% | **Total direct costs, mean, PPPY**  $31,342  **Key direct costs, mean, PPPY**  All-cause medical costs: $22,790  -Proportion epilepsy related costs: 27.3%  Pharmacy costs: $8551  -Proportion ASM costs: 62.8% | *€31,342  $31,342 | NR | Probable DS is  associated with substantial medical and pharmacy costs for US Medicaid plans. |
| Lagae 2019 [20]  Full | EU5 (France, Germany, Italy, Spain, UK) | DISCUSS EU5 cohort  Online survey. Caregivers of patients with DS answered questions related to the previous 3 months | NR | Inclusion year: 2016  Cost year: 2016 | N=584 caregivers of paediatric (83%) and adult (17%) patients with DS | **Total direct costs, mean, PPPY**  $15,885  **Key direct costs, mean, PPPY**  Seizure-related symptoms:  $7957  - ED: $587  -Ambulance calls: $774  -Epilepsy specialist: $493  -ASMs: $6103 (38% of total cost)  Non-seizure-related symptoms (e.g physiotherapy and therapies for speech, ADHD, ASD, learning and behaviour): $7929 (50% of cost) | *€15,885  $15,885 | NR | Drivers of total direct cost are ASMs (38%) and non-seizure related therapies (50%).  Costs were higher for patients in the highest than lowest seizure burden group for:  emergency admissions, ambulance calls, epilepsy specialist visits and physiotherapy |
| Reaven 2019 [29]  Full | USA | Retrospective insurance claims analysis: Truven Health Analytics MarketScan®  Research Databases, entries for ~60 million people with commercial or Medicaid insurance coverage | Probable DS:   - No diagnoses atypical in DS - No repeated use of drugs generally contraindicated in DS - Either prescriptions for ≥2 distinct ASMs - filled within 90 days of the index date   or a diagnosis of febrile seizures (ICD-9780.31 or 780.32) | Inclusion year: 2010–2015  Cost year 2017 | Commercial:  N=321  Mean age: 12 (10.8 [0–62])  Male: 55%  Medicaid:  N=668  Mean age: 17 (15.5 [0–69])  Male: 56.9% | *Commercial:* **Total direct costs, mean (SD), PPPY**  All patients: $31,433 (41,835)  With events vs no event: $43,758 vs $20,208  **Key direct costs, mean (SD), PPPY**  Total services: $24,376 (38,308)  Total drugs: $7057 (9962)  Inpatient admissions: $10,847 (28,133) (34.5%)  ED visits: $1811 (3290)  Hospital OP visits: $5769 (11,643)  Physician visits: $2154 (3255)  Other OP: $1817 (4479)  Home health: $1540 (8445)  ASMs: $4130 (5375)  *Medicaid:* **Total direct costs, mean (SD), PPPY**  All patients: $31,884 (45,174)  With events vs no event: $31,278 vs $32,650  **Key direct costs, mean (SD), PPPY**  Total services: $27,039 (42,876)  Total drugs: $4845 (6904)  Inpatient admissions: $5694 (23,135) (17.8%)  ED visits: $810 (1647)  Hospital OP visits: $1728 (1647)  Physician visits: $574 (961)  Other OP: $7566 (20,646)  Home health: $10,390 (26,767)  ASMs: $2488 (4440) | *Commercial*  *€31,433  $31,433  *Medicaid:*  *€31,884  $31,884 | NR | Patients with DS having at least one medically treated seizure event incur  substantial all-cause costs |
| Schubert-Bast 2022 [34]  Full | Germany | Retrospective insurance claims analysis: Vilua Healthcare research database, entries for >4 million people i.e ~5% of the German population | Probable DS:   - ≥1 ICD-10 diagnosis of G40 (epilepsy)/G41 (SE) and ≥1 prescription of stiripentol, potassium bromide, or VPA+CLB+other ASMs (identified in the context of the epilepsy diagnosis) - Exclusion of any other identified epilepsy syndrome, abnormal brain development, or prescription of Na+ channel blockers | Inclusion year: 2007–2016  Cost year: 2015 | N=160 children & adults with probable DS over 10-year period  N=64 identified in 2016  Mean age 33.2 (range: 3–82) years; 48% male | **Total direct costs, mean PPPY**  €11,048  **Key direct costs, mean PPPY (%)**  Inpatient: €5147 (47%)  Medication: €2826 (26%)  ASMs: €1043 (37% of medication)  Services and devices: €2044 (19%) | €11,048  *$11,048 | NR | Cost drivers were inpatient care (47%), medication (26%), and services and devices (19%).  Costs were significantly higher in patients with rescue medication vs without |
| Stockl 2019 [38]  Abstract | USA | Retrospective insurance claims analysis: IBM® MarketScan® Commercial & Medicaid databases | Probable DS (LGS or DRE)   - ≥1 ASM claim and medical claims with ≥1 diagnosis code for LGS or refractory epilepsy or ≥1 claim for clobazam or rufinamide. | Inclusion year: 2015–2016  Cost year: NR | Probable DS (N=183)  Mean age: 14.5 years | *Commercial:* Probable DS  **Total direct costs, mean PPPY**  $77,914  **Key direct costs, mean PPPY**  All-cause medical costs: $63,850  -Proportion epilepsy related costs: 80.5%  Pharmacy costs: $14,064  -Proportion ASM costs: 70.8% | *€77,914  $77,914 | NR | The majority of medical costs were epilepsy-related (71.2%, 80.5%, and 62.5%, respectively).  The majority of pharmacy costs were for ASMs (72.6%, 70.8%, 65.8%, respectively). |
| Strzelczyk 2014 [40]  Full | Germany | Retrospective, single centre study of patients with DS | Clinical diagnosis of DS | Inclusion year: 2007–2010  Cost year: 2011 | N=13  Mean age: 12.3 (7.5) (median: 11, range: 3–28 years)  Male: 31% | **Epilepsy specific total direct costs, mean (SD), PPPY**  €6506 (3974)  **Key direct costs, mean (SD) (%), PPPY**  Hospitalisation: €4483 (3684) (68.9%)  ASM: €1559 (1356) (24.0%) | NR | NR | The major cost driver was  hospitalization (68.9% of total direct costs), ahead of costs for anticonvulsants (24.0%)  Direct costs of patients with DS were above the average European costs of drug-resistant epilepsy in children. |
| Strzelczyk 2019 [41] | Germany | Cross-sectional, prospective  multicenter study; carers of patients with DS completed surveys spanning previous 3 months (German DS study) | Patients with DS and their caregivers recruited from throughout centres in Germany  and through the German DS patient advocacy  group | Inclusion year: 2017–2018  Cost year: 2017 | N=93  Mean age: 10.1 (7.1) years (median: 8.7, range: 15 months–33.7 years)  Male: 53% | **Total direct costs, mean (SD) PPP 3-months**  €6,043 (5,825)  **Key direct costs, mean (SD) PPP 3-months)**  Inpatient costs: €1,702 (4315) (28%)  Care grade (informal care) benefits: €1130 (805) (19%)  ASM costs: €892 (1017) (15%)  Ancillary treatments: €559 (503) (9%) | €24,172  *$24,172  (Calculated €6,043 x 4) | **Total indirect costs (lost productivity), mean (SD) PPP 3-months**  Maternal: €4399 (4989)  Paternal: €391 (1,352)  *Total: €4790  PPPY:  Maternal: €17,596  Paternal: €1564  Total: €19,160 | Inpatient costs formed the single most important  category of direct health care cost  In a univariate  analysis seizure frequency, experience of SE, nursing care level and severe additional symptoms were associated with total direct healthcare costs. |
| Strzelczyk 2019 [42]  Full | Germany | DS vs DRE vs SR  German DS study & the EpiPaed study (completed in 2011 by 489  caregivers of children with epilepsy | Patients with DS and their caregivers recruited from throughout centres in Germany  and through the German DS patient advocacy  group | Inclusion year: 2018  Cost year: 2017 | Paediatric DS vs DRE vs SR  Mean age: 8.1 vs 7.6 vs 8.3 years  Male: 55 vs 54 vs 57%  Adult DS vs DRE vs SR  Mean age: 24.6 vs 23.9 vs 23.6 years  Male: 46 vs 46 vs 46% | **Total direct costs mean (SD) PPP 3-months**  DS vs DRE vs SR  €4864 (€4995) vs €3049 (€5022) P = 0.01 vs €1007 (€1729) P < 0.001  **Key direct costs, mean (SD) PPP 3-months)**  Inpatient costs: €1567.8 (32.2%) vs €1794.0 vs €214.4  Care grade costs: €1129.6 vs €414.5 vs €349.2  ASM costs: €892.2 vs €348.6 vs €116.5  Ancillary treatments: €477.7 vs €116.0 vs €112.5 | €19,456  *$19,456  (Calculated €4864 x 4) | **Total indirect costs (lost productivity), mean (SD) PPP 3-months**  DS vs DRE vs SR  €4757 vs €1541 (P < 0.001) vs €891 (P < 0.001)  PPPY in DS:  €4757x4=€19,028 | Care grade and inpatient direct costs are highest for DS and patients with drug-resistant epilepsy  More caregivers of patients with DS gave up work, and mean indirect productivity costs were highest for the DS cohort  Seizure frequency had a significant impact on health care resource use. |
| Strzelczyk 2022 [43]  Full | Germany | Carers of patients with DS completed surveys  (German results from DISCUSS study) | NR | NR | N=68  Mean age: 10 years (median: 9, range: 1-26).  Male: 52.9% | Seizure-related symptoms (emergency treatment, specialist visits, medication), PPPY: €7,892  Comorbidities, PPPY: €7,271 | NR | NR | The costs of treating a patient with DS are high |
| Whittington 2018 [48]  Full | USA | Survey of caregivers of patients with DS treated at Children's Hospital Colorado | NR | Inclusion year: 2010–2014  Cost year: 2016 | N=34 caregivers of patients with DS  NR | **Total direct costs, mean PPPY**  $27,000 (95% CI: $15,757, $41,904)  **Key direct costs, mean PPPY (SD) (%)**  Hospitalisations: $11,565 ($22,001) (43%)  In-home medical care visits: $9894 ($29,456) (37%) | *€27,000  $27,000 | Lost productivity, income & leisure time: $81,582  (95% CI: $57,253, $110,151)  Lost productivity: $19,925 | DS is associated with considerable healthcare utilization, financial burden, and time commitment. |

ASM, anti-seizure medication; CI, confidence interval; DS, Dravet syndrome; DRE, drug-resistant epilepsy; ED, emergency department; NR, not reported; OP, outpatient; PPPY, per person per year; SD, standard deviation; SE, status epilepticus; SR, seizure remission

Google Finance 30^th^ August: 1.00 US Dollar =1 EUR

**Supplemental Table S5: Resource**

| **Study** | **Country** | **Study type, source of data** | **Definition of DS** | **Year** | **Population (n); age/% male** | **Healthcare resource utilisation** | **Length of stay (LOS)** | **Anti-seizure medication (ASM) use** | **Notes** |
| --- | --- | --- | --- | --- | --- | --- | --- | --- | --- |
| Aledo-Serrano 2020 [1]  Full | Spain | The Dravet syndrome caregiver on-line survey (DISCUSS survey)- Spanish cohort | The Dravet Syndrome Foundation in Spain recruited by invitation a cohort of 57 caregivers of Spanish patients diagnosed with DS | 2016 | N=57  Mean (SD) age: 9.13 (6.88)  Male: 49% | % of patients in last year:  Hospitalisation: 60%  Ambulance: 40% |  |  | In the cases in which the diagnosis was delayed between one and two years, 30% of the patients were exposed to one or two contraindicated drugs. |
| Aras 2015 [2]  Full | 15 European countries (highest number of responses from Spain, Netherlands, Italy, Germany, and France) | Web-based survey of caregivers of patients with DS administered by the Dravet Syndrome  Foundation Spain. | Patients with DS  identified by patient organizations through their affiliated distribution  lists. | 2014 | N=274  Age range: 1–47 years (N=104 [38%] aged 4–8 years)  Male: 57% | Admission to ED due to SE (% patients):  0: 67%  1: 15%  ≤2: 18% | NR | Proportion of patients (%):  3 ASMs: 40%  4 ASMs: 25%  VPA: 86%  CLB: 55%  TPM: 44%  STP: 42% | Over a third of patients with Dravet syndrome had taken sodium channel blockers in the past |
| Gara-Adams 2021 [13]  Abstract | England, UK | Interviews with clinicians directly involved with the care of patients with DS (N=18) | NA | NR | NA | Emergency resource use after rescue medication:  Ambulance called (% of patients):  Paediatric: 26.7% (14–18 years) to 82.3% (2–3 years)  Adults: 26.5% (18–25 years) to 24.5% (≥ 18 years)  % admitted from A&E to:  Hospital  Paediatric: 21.7% (14–18 years) to 55.0% (2–3 years)  Adults: 71.0% (18–25 years and ≥ 18 years)  ICU  Paediatric: 2.3% (14–18 years) to 18.0% (2–3 years)  Adults: 12.0% (18–25 years and ≥ 18 years) | NR | NR | High seizure frequency was associated with much higher resource use compared to medium and low seizure frequency groups, especially for secondary care and nurse non-face-to-face consultations (telephone and email) in younger age groups |
| Huang 2021 [17]  Full | Taiwan | On-line questionnaire of caregivers of patients with a diagnosis of DS (diagnosed and actively followed up by a paediatric neurologist in Taiwan. | Diagnosed and actively followed up by a paediatric neurologist in Taiwan. All patients had a confirmed mutation in *SCN1A*. | 2019/2020 | N=38  Mean age (SD): 10.5 (2.6 [range: 1–28]) years  Male: 58% | NR | NR | Proportion of patients (%):  1 ASM: 5%  2 ASMs: 16%  > 3 ASMs: 78%  CLB: 68%  VPA: 66%  LEV: 55%  TPM: 29% STP: 26% | Patients with DS required multiple ASMs for seizure control, with and 78% taking > 3 drugs  Contraindicated medications were reported including lamotrigine (11%), carbamazepine (3%), and  oxcarbazepine (24%). |
| Hollenack 2019 [16]  Abstract | USA | Retrospective insurance claims analysis: IBM® MarketScan® Medicaid databases | Probable DS (LGS or DRE)   - ≥1 ASM claim and medical claims with ≥1 diagnosis code for LGS or refractory epilepsy or ≥1 claim for clobazam or rufinamide. | 2014–2015 | Probable DS (N=504)  Mean age: 17.7 years  Male: 54.8% | NR | NR | Average number of distinct ASMs during the 12-month pre-index period: 2.3 | - |
| Lagae 2018 [19]  Full | Mainly Europe, especially Italy, the UK, Germany, France, the Netherlands, Spain, and Poland (~15% of DS patients in Europe) | The Dravet syndrome caregiver on-line survey (DISCUSS survey) | Participants recruited through e-mail invitations to approximately 1000 members of different countries’ patient advocacy groups associated with the Dravet Syndrome European Federation, as well as through Internet-based sources (Facebook and Twitter). | 2016 | N=584 caregivers of paediatric (83%) and adult (17%) patients with DS  Mean age: 10.6 years  Male patients: 52% | Events in the past 12 months (% of patients):  ED: 50.3%  Ambulance calls: 46.1% | NR | Current  Mean (SD [range]): 3.14 (1.3; [0–12])  Previous: 3.35  (3.5 [0–28])  Proportion of patients (%):  VPA: 76%  CLB: 53%  STP: 47%  TPM: 34% | Very few (0–2%) patients reported currently taking  antiepileptic agents known to exacerbate seizures in DS; however, many adults had taken these previously, including carbamazepine (54%), lamotrigine (56%),  phenobarbital (42%), and vigabatrin (35%)  The use of pharmacological treatments for the associated  comorbidities appeared limited.  High seizure frequency was related to more  reports of emergency treatment, comorbidities, and a lower QoL (as measured by the  standardized instrument EQ-5D-5L). |
| Lagae 2019 [20]  Full | EU5 (France, Germany, Italy, Spain, UK) | DISCUSS EU5 cohort  Online survey. Caregivers of patients with DS answered questions related to the previous 3 months | NR | 2016 | N=584 caregivers of paediatric (83%) and adult (17%) patients with DS | High vs low seizure burden  Mean (SD) annual number  ED: 3.6 (5.4) vs 2.0  (4.1); P < 0.001  Ambulance calls: 3.3  (5.0) vs 1.2 (2.3); P < 0.05  Epilepsy specialist: 6.0  (5.4) vs 2.7 (3.2); P < 0.001  Physiotherapy: 36.1  (39.7) vs 18.6 (31.9); P < 0.001  Speech therapy: 22.6 (32.8) vs 26.4 (36.6)  Therapy for learning difficulties: 12.7 (30.7) vs 18.3 (33.8)  Autism therapy: 4.9  (18.4) vs 4.8 (18.2)  ADHD therapy: 1.0 (8.4) vs 1.1 (9.9)  Behavioural therapy: 5.0  (18.6) vs 6.4 (19.6) | NR | NR | Healthcare resource use for epilepsy-related symptoms in the highest  seizure subgroup was  significantly higher than in the lowest seizure frequency group  for number of epilepsy specialist visits, emergency admissions, ambulance calls and physiotherapy visits (P < 0.001, P < 0.001, P < 0.05; P < 0.001, respectively) |
| Owen-Pickrell 2022 [26]  Full | UK | Retrospective analysis of EMR from healthcare databases: Primary care data (CPRD) linked to secondary care data (HES), and general population mortality data (ONS) (13.7 million de-identified EMRs of children & adults) | Confirmed DS: DS Read Codes (F25G.11 or F25G.00)  Probable DS: ICD-10/CRPD Read Code for epilepsy plus prescription for stiripentol or potassium bromide | 1987–2018 | Overall: N=54  Confirmed DS: N=32  Probable DS: N=22  Mean age 7.6 (SD: 10.9); median 3.0 (range: 0.0; 45.0); 50% male | Mean (SD) < 12 years vs ≥ 12 years (PPPY)  *Confirmed DS*  Primary care consultations: 6.50 (4.55) vs 7.50 (6.47)  Hospital outpatient visits: 8.3 (7.39) vs 6.43 (6.32)  Hospital inpatient admissions:  -All cause: 1.00 (1.25) vs 0.86 (1.46)  - Epilepsy-related: 0.90 (1.10) vs 0.71 (1.11)  ED visits: 0.90 (1.60) vs 0.29 (0.76)  *Probable DS*  Primary care consultations: 9.59 (5.99) vs 9.13  (10.78)  Hospital outpatient visits: 7.58 (3.90) vs 5.6 (4.38)  Hospital inpatient admissions:  -All cause: 4.00 (3.81) vs 1.70 (2.75)  - Epilepsy-related: 3.33 (3.55) vs 1.70 (2.75)  ED visits: 2.33 (2.50) vs 1.60 (2.55) | Mean (SD), days  *Confirmed DS*  All causes: 1.00 (1.67) vs 0.63 (1.54)  Epilepsy-related: 1.14 (1.77) vs 0.71 (1.64)  *Probable DS*  All causes: 1.28 (2.35) vs 2.16 (4.52)  Epilepsy-related: 1.16 (2.21) vs  2.15 (4.55) | *Confirmed DS*  Mean (SD) number of ASMs during the follow-up (3.4 years); 5.5 (2.7; [1–12])    Midazolam: 88%  VPA: 81%  CLB: 72%  *Probable DS*  Mean (SD; [range]) number of ASMs during the follow-up (10 years): 7.6 (3.8;  [3–15])  STP: 91%  VPA: 86%  CLB: 86% | A greater proportion of patients with probable DS than confirmed DS were prescribed  sodium channel blockers, such as lamotrigine and carbamazepine, that  are contraindicated for DS  The number of  hospital admissions in patients with probable DS <12 years was  particularly high; this may reflect more severe seizures in younger patients due to natural DS progression misdiagnosis for focal epilepsy  and therefore incorrectly prescribed ASMs (such as carbamazepine) that  can exacerbate seizures in children with DS, and the time taken to  achieve the right ASM regime |
| Pagano 2019 [27]  Abstract | UK | The Dravet syndrome caregiver on-line survey (DISCUSS survey)- Spanish cohort | Identified through patient organisations | 2016 | N=72  caregivers of paediatric (78%) and adult (22%) patients with DS  Mean age: 11 (range: <1–34) years | Mean paediatric neurology visits: 3 PPPY  Emergency admission and ambulance call: 60% of patients | NR | NR | - |
| Reaven 2019 [29]  Full | USA | Retrospective insurance claims analysis: Truven Health Analytics MarketScan®  Research Databases, entries for ~60 million people with commercial or Medicaid insurance coverage | Probable DS:  No diagnoses atypical in DS  No repeated use of drugs generally contraindicated in DS  Either prescriptions for ≥2 distinct ASMs  filled within 90 days of the index date or a diagnosis of febrile seizures (ICD-9780.31 or 780.32) | 2010–2015  Cost year 2017 | Commercial:  N=321  Mean age: 12 (10.8 [0–62])  Male: 55%  Medicaid:  N=668  Mean age: 17 (15.5 [0–69])  Male: 56.9% | Mean (SD), PPPY  *Commercial:*  Inpatient admissions: 0.5 (0.8)  ED visits: 1.0 (1.5)  Hospital OP visits: 7.0 (20.3)  Physician visits: 10.7 (11.5)  Other OP: 9.1 (20.1)  Home health: 5.7 (20.0)  Equipment/ supply: 1.0 (3.0)  ASMs: 18.9 (11.5)  Rescue ASMs: 1.0 (3.2)  Other drugs: 15.0 (16.3)    *Medicaid:*  Inpatient admissions: 0.4 (0.7)  ED visits: 1.4 (2.1)  Hospital OP visits: 3.8 (8.3)  Physician visits: 6.4 (9.4)  Other OP: 43.1 (84.2)  Home health: 44.4 (97.8)  Equipment/ supply: 0.7 (2.7)  ASMs: 21.3 (13.1)  Rescue ASMs: 1.0 (2.4)  Other drugs: 27.7 (35.9) | NR | NR | Patients with DS with at least one  medically treated seizure event utilized high levels of healthcare services and drugs and had substantial all-cause costs |
| Schubert-Bast 2022 [34]  Full | Germany | Retrospective insurance claims analysis: Vilua Healthcare research database, entries for >4 million people i.e ~5% of the German population | Probable DS:  ≥1 ICD-10 diagnosis of G40 (epilepsy)/G41 (SE) and ≥1 prescription of stiripentol, potassium bromide, or VPA+CLB+other ASMs (identified in the context of the epilepsy diagnosis)  Exclusion of any other identified epilepsy syndrome, abnormal brain development, or prescription of Na+ channel blockers | 2007–2016  Cost year: 2015 | N=160 children & adults with probable DS over 10-year period  N=64 identified in 2016  Mean age 33.2 (range: 3–82) years  Male: 48% | AHR, mean (SD [range])  1.1 (1.7 [0–14]) | Mean (SD, [range])  17.5 (33.5 [0–236 days]) | Mean (SD [range])  2.6 (1.2 [1–7]) PPPY  5.0 (2.5 [1–9]) over the entire observable time for each patient | Costs, resource use, AHR and LOS were significantly higher in patients with rescue medication vs without |
| Stockl 2019 [39] | USA | Retrospective insurance claims analysis: Truven Health Analytics MarketScan®  Research Databases, entries for ~60 million people with commercial or Medicaid insurance coverage | ≥ 1 ASM and ≥ diagnostic code for DRE  patients with an acute inpatient hospitalization (≥1 day LOS) |  | Probable DS:  *Commercial* (N=174), *Medicaid* (N=303) | Hospitalisations by healthplan (% of patients):  Epilepsy-related index-hospitalizations: 63–70%  Pneumonia-related index hospitalizations: 2–3%  Injury-related index-hospitalizations: 2%*  ICU use: 31%*  * Data are for all patients (LGS+DS+TSC) | Mean (SD) LOS ICU vs non-ICU use: 8.0 (16.8) vs 4.0 (7.9) days*  * Data are for all patients (LGS+DS+TSC) | NR | Epilepsy was the major cause of hospitalization in commercial and Medicaid plans. |
| Stockl 2019 [38]  Abstract | USA | Retrospective insurance claims analysis: IBM® MarketScan® Commercial & Medicaid databases | Probable DS (LGS or DRE)  ≥1 ASM claim and medical claims with ≥1 diagnosis code for LGS or refractory epilepsy or ≥1 claim for clobazam or rufinamide. | 2015–2016 | Probable DS (N=183)  Mean age: 14.5 years | NR | NR | Average number of distinct ASMs during the 12-month pre-index period: 2.6 | - |
| Strzelczyk 2014 [40]  Full | Germany | Retrospective, single centre study of patients with DS | Clinical diagnosis of DS | 2007–2010  Cost year: 2011 | N=13  Mean age: 12.3 (7.5) (median: 11, range: 3–28 years)  Male: 31% | NR | NR | Mean (SD): 2.4  (0.7, range: 1–3).  VPA: 84.6%  TPM: 76.9% Bromide: 38.5% | - |
| Strzelczyk 2019 [41]  Full | Germany | Cross-sectional, prospective  multicenter study; carers of patients with DS completed surveys  (German DS study) | Patients with DS and their caregivers recruited from throughout centres in Germany  and through the German DS patient advocacy  group | Cost year: 2017 | N=93  Mean age: 10.1 (7.1) years (median: 8.7, range: 15 months–33.7 years)  Male: 53% | Annual resource use (% of patients):  Inpatient: 52%  ED: 47%  ICU: 22%  AHR, mean (SD [range])  4.3 (5.3 [1–35]) | N=48 hospitalized patients  LOS, mean (SD, [range])  25.6 (39.6 [1–200 days])  ICU LOS, mean (SD, [range])  5.5 (5.9 [1–23 days]) | ASM use, mean (SD [range])  2.5 (1.1 [0–6])  VPA: 66%  Potassium bromide: 44%  CLB: 41%  STP: 35%  TPM: 24% |  |
| Strzelczyk 2019 [42]  Full | Germany | DS vs DRE vs SR  German DS study & the EpiPaed study (completed in 2011 by 489  caregivers of children with epilepsy) | Patients with DS and their caregivers recruited from throughout centres in Germany  and through the German DS patient advocacy  group | Cost year: 2017 | DS vs DRE vs SR  N= 93 vs 93 vs 93  Paediatric DS vs DRE vs SR  Mean age: 8.1 vs 7.6 vs 8.3 years  Male: 55 vs 54 vs 57%  Adult DS vs DRE vs SR  Mean age: 24.6 vs 23.9 vs 23.6 years  Male: 46 vs 46 vs 46% | NR | Mean LOS, days:  DS vs DRE vs SR  2.5 vs 2.9 vs 0.3 days; P = 0.002 in both comparisons). | Mean number of ASMs  DS vs DRE vs SR  2.2 vs 1.4 vs 1.0 (P < 0.001 in both comparisons)  VPA: 66%  Potassium bromide: 44%  CLB: 41%  STP: 35%  TPM: 24% | Patients with DS used more ASMs compared  with DRE and SR patients.  Seizure frequency was found to have a significant impact on health care resource use, with each categorical increase in seizure frequency corresponding to an average €730 increment. Patient age and parental BDI‐II scores also significantly impacted health care resource use. |
| Strzelczyk 2022 [43]  Full | Germany | Carers of patients with DS completed surveys  (German results from DISCUSS study) | NR |  | N=68  Mean age: 10 years (median: 9, range: 1-26).  Male: 52.9% | NR | NR | Mean (SD [range])  Current: 3 (1.4 [0-6])  Past: 3.2 (3.0 [0–11])  VPA: 63.2%  Potassium bromide: 48.5%  STP: 44.1%  CLB: 39.7% TPM: 23.5% | - |

AHR, annual hospitalisation rate; ASM, anti-seizure medication; A&E, accident and emergency; CLB, clobazam; DS, Dravet syndrome; DRE, drug-resistant epilepsy; ED, emergency department; ICU, intensive care unit; LEV, levetiracetam; LGS, Lennox-Gastaut syndrome; NA, non-applicable; NR, not reported; OP, outpatient; SD, standard deviation; SE, status epilepticus; SR, seizure remission; STP, stiripentol; TSC, tuberous sclerosis complex; TPM, topiramate; VPA, valproic acid

**Supplemental Table S6: Quality assessment checklist for epidemiology studies**

|  | **Hurst 1990 [18] Full** | **Brunklaus 2012 [7] Full** | **Bayat 2015 [4] Full** | **Rosander 2015 [30] Full** | **Wu 2015 [49];**  **Full** | **Hollenack 2019 [15]**  **Abstract** | **Symonds 2021 [45] Abstract** | **Owen-Pickrell 2022 [26] Full** | **Schubert-Bast 2022 [34]**  **Full** | **Bjurulf 2022 [5]**  **Full** |
| --- | --- | --- | --- | --- | --- | --- | --- | --- | --- | --- |
| Outcome | Incidence | Incidence | Incidence | Incidence & prevalence | Incidence | Prevalence | Incidence | Prevalence | Prevalence | Incidence & prevalence |
| Was the study’s target population a close representation of the national population in relation to relevant variables, e.g. age, sex, occupation? | 2 | 0 | 0 | 0 | 1 | 0 | 1 | 0 | 0 | 0 |
| Was the sampling frame a true or close representation of the target population? | 1 | 0 | 0 | 0 | 1 | 0 | 1 | 0 | 0 | 0 |
| Was some form of random selection used to select the sample, OR, was a census undertaken? | 2 | 0 | 0 | 0 | 1 | 0 | 1 | 0 | 0 | 0 |
| Was the likelihood of non-response bias minimal? | 1 | 1 | 1 | 1 | 1 | 1 | 1 | 1 | 1 | 1 |
| Were data collected directly from the subjects (as opposed to a proxy)? | 1 | 1 | 1 | 1 | 1 | 1 | 1 | 0 | 1 | 1 |
| Was an acceptable case definition used in the study? | 1 | 1 | 1 | 1 | 1 | 1 | 1 | 1 | 1 | 1 |
| Was the study instrument that measured the parameter of interest (e.g. prevalence of low back pain) shown to have reliability and validity (if necessary)? | 1 | 1 | 1 | 1 | 1 | 2 | 1 | 1 | 1 | 1 |
| Was the same mode of data collection used for all subjects? | 0 | 0 | 0 | 0 | 0 | 0 | 0 | 0 | 0 | 0 |
| Were the numerator(s) and denominator(s) for the parameter of interest appropriate? | 1 | 1 | 1 | 1 | 1 | 1 | 1 | 1 | 1 | 1 |
| Overall risk of bias  Low risk=0-6  Moderate =7-12  High =13-18 | 10=Moderate | 5= Low | 5= Low | 5= Low | 8=Moderate | 6=Low | 8=Moderate | 4=Low | 5= Low | 5=Low |

From Hoy et al [50]; 0=low risk; 1= moderate/unclear; 2=high risk

**Supplemental Table S7**: **Quality assessment checklist for qualitative HRQoL studies**

|  | **Appraisal/score** | **Aledo-Serrano 2020 [1]**  **Spain**  **Full** | **Huang 2021 [17]**  **Full** | **Nabbout 2018 [22]**  **Full** | **Nabbout 2019 [23]**  **Full** | **Nabbout 2020 [24]**  **Full** | **Nolan 2006 [25]**  **Full** | **Villas 2017 [47]**  **Full** |
| --- | --- | --- | --- | --- | --- | --- | --- | --- |
| Addresses a research question closely related to our review aims | Yes/No  Only “yes” can be grade I or II | Yes | Yes | Yes | Yes | Yes | Yes | Yes |
| Qualitative methods are appropriate for the research question | Yes=3  No=0 | 3 | 3 | 3 | 3 | 3 | 3 | 3 |
| Details of caregiver (relationship to patient, age, gender) and patient features (age, seizure frequency/ disease severity) reported | Both caregiver and patient = 3  Only one = 1  Neither = 0 | 3 | 1 | 1 | 1 | 1 | 1 | 1 |
| Methods described in sufficient detail (e.g., how participants were recruited, what did the interview guide ask, etc.) | Yes = 3  Partial = 1  No = 0 | 1 | 1 | 1 | 1 | 1 | 1 | 3 |
| Analysis described in sufficient detail (analysis approach e.g., grounded theory/thematic analysis, analysis procedures, saturation assessed) [yes/no score: 0/2] | Yes = 3  Partial = 1  No = 0 | 1 | 1 | 1 | 1 | 1 | 1 | 1 |
| Caregiver quotes included | Yes = 1  No = 0 | 0 | 0 | 1 | 1 | 0 | 0 | 0 |
| Reports ethical review/approval | Yes = 1  No = 0 | 0 | 1 | 1 | 1 | 1 | 1 | 1 |
| Evidence of obvious bias in methodology (e.g., recruitment bias, focused on one treatment) | No obvious bias = 3  Some evidence of bias = 1  Several sources of bias = 0 | 1 | 1 | 1 | 1 | 1 | 1 | 1 |
| Total score range | 13–17 Grade I (if ‘yes’ to first question)  8–12 Grade II  0–7 Grade III | 9=Grade 2 | 8=Grade 2 | 9=Grade 2 | 9=Grade 2 | 8=Grade 2 | 8=Grade 2 | 10=Grade 2 |

From Gallop et al [51] Grade I= highest methodological and reporting quality; Grade 2= moderate-high methodological and reporting quality; Grade 3= limitations in their methodological and reporting quality

**Supplemental Table S8: HRQoL: Quality assessment checklist for quantitative HRQoL studies**

|  | **Appraisal/score** | **Auvin 2021 [3]**  **Full** | **Brunklaus 2011 [6]**  **Full** | **Campbell 2018 [9]**  **Full** | **deLange 2019 [12]** | **Lagae 2018 [19]**  **Europe**  **Full &**  **Lagae 2019 [20]**  **Full (DISCUSS)** | **Lo 2021**  **Full [21]** | **Pagano 2019 [27]**  **Abstract**  **(DISCUSS UK)** | **Paprocka 2021 [28]**  **Full** | **Schoonjans 2019 [33]**  **Full** | **Sinoo 2019 [35]**  **Full** | **Steckler 2020 [37]**  **Abstract** | **Strzelczyk 2019 [41]**  **& Strzelczyk 2019 [42] &**  **Strzelczyk 2022 [44]** | **Strzelczyk 2022 [43]**  **Full**  **(DISCUSS Germany)** |
| --- | --- | --- | --- | --- | --- | --- | --- | --- | --- | --- | --- | --- | --- | --- |
| Addresses a research question closely related to our review aims | Yes/No  Only “yes” can be grade I or II | Yes | Yes | Yes | Yes | Yes | Yes | Yes | Yes | Yes | Yes | Yes | Yes | Yes |
| Validated questionnaires (e.g., EQ-5D, ZBI, CarerQol, SF-36, GAD, BDI) | Validated questionnaires = 3  Well-described bespoke survey = 1  Poorly described survey = 0 | 1 | 3 | 3 | 3 | 3 | 1 | 3 | 3 | 3 | 3 | 3 | 3 | 3 |
| Good sample size | 50+=3  25–50=1  Less than 25=0 | 3 | 3 | 1 | 3 | 3 | 3 | 3 | 3 | 3 | 3 | 3 | 3 | 3 |
| Details of caregiver (relationship to patient, age, gender) and patient features (age, seizure frequency/ disease severity) reported | Both caregiver and patient = 3  Only one = 1  Neither = 0 | 1 | 1 | 1 | 1 | 1 | 1 | 0 | 1 | 1 | 1 | 0 | 3 | 1 |
| Appropriate statistical reporting | Yes = 1  No = 0 | 1 | 1 | 1 | 1 | 1 | 1 | 1 | 1 | 1 | 1 | 1 | 1 | 1 |
| Reports ethical review/approval | Yes = 1  No = 0 | 1 | 1 | 1 | 1 | 1 | 0 | 0 | 1 | 1 | 1 | 0 | 1 | 1 |
| Evidence of obvious bias in methodology (e.g., recruitment bias, focused on one treatment) | No obvious bias = 3  Some evidence of bias = 1  Several sources of bias = 0 | 1 | 1 | 1 | 1 | 1 | 1 | 1 | 1 | 1 | 1 | 1 | 1 | 1 |
| Total score range | 11–14 Grade I (if ‘yes’ to first question)  7–10 Grade II  0–6 Grade III | 8: Grade II | 10: Grade II | 8: Grade II | 10: Grade II | 10: Grade II | 7: Grade II | 8: Grade II | 10: Grade II | 10: Grade II | 10: Grade II | 8: Grade II | 12: Grade I | 10: Grade II |

From Gallop et al [51] Grade I= highest methodological and reporting quality; Grade 2= moderate-high methodological and reporting quality; Grade 3= limitations in their methodological and reporting quality

**Supplemental Table S9: Quality assessment checklist of cost-of-illness studies**

|  | **Ceska 2021[10]**  **Full** | **Hollenack 2019 [16]**  **Abstract** | **Lagae 2018 [19]**  **Full & Lagae 2019 [20]**  **Full**  **(DISCUSS)** | **Owen-Pickrell 2022 [26]**  **Full** | **Reaven 2019 [29]**  **Full** | **Stockl 2019 [39] & Stockl 2019 [38]**  **Abstract** | **Schubert-Bast 2022 [34]**  **Full** | **Strzelczyk 2019 [41]**  **& Strzelczyk 2019 [42] Full** | **Strzelczyk 2014 [40]**  **Full** | **Strzelczyk 2022 [43]**  **Full**  **(DISCUSS Germany)** | **Whittington 2018 [48]**  **Full** |
| --- | --- | --- | --- | --- | --- | --- | --- | --- | --- | --- | --- |
| Was a clear definition of the illness given? | P | P | P | P | P | P | P | P | P | P | P |
| Were epidemiological sources carefully described? | 1 | 1 | 1 | 1 | 1 | 1 | 1 | 1 | 1 | 1 | 1 |
| Were direct/indirect costs/ resource sufficiently disaggregated? | P | P | 1 | NA | P | P | P | 1 | P | P | 1 |
| Were activity data sources carefully described? | P | P | 1 | 1 | 1 | P | 1 | 1 | P | 1 | 1 |
| Were activity data appropriately assessed? | 0 (only 12 pts) | P | 1 | 1 | 1 | P | 1 | 1 | 0 (only 13 pts) | P | P (only one centre) |
| Were the sources of all cost/HCRU values analytically described? | P | 0 | P | P | P | 0 | P | 1 | P | P | P |
| Were unit costs appropriately valued? | P | 1 | 1 | NA | 1 | 1 | 1 | 1 | P | 1 | 1 |
| Were the methods adopted carefully explained? | 1 | 0 | 1 | 1 | 1 | 0 | 1 | 1 | 1 | 1 | 1 |
| Were the major assumptions tested in a sensitivity analysis? | 0 | 0 | 0 | 0 | 0 | 0 | 0 | 0 | 0 | 0 | 0 |
| Was the presentation of study results consistent with the methodology of the study? | 1 | 1 | 1 | 1 | 1 | 1 | 1 | 1 | 1 | 1 | 1 |
| Total score by study |  |  |  |  |  |  |  |  |  |  |  |
| YES(1)=low risk | 3 | 3 | **7** | **5** | **6** | 3 | **6** | **8** | 3 | **5** | **6** |
| NO(0)=high risk | 2 | 3 | 1 | 1 | 1 | 3 | 1 | 1 | 2 | 1 | 1 |
| PARTIALLY(p)=moderate risk | **5** | **4** | 2 | 2 | 3 | **4** | 3 | 1 | **5** | 4 | 3 |

From Molinier et al.[52] Mostly1=low risk; P= moderate/unclear; 0=high risk; NA=non-applicable (HCRU studies)

References

1. Aledo-Serrano A, Mingorance A. Analysis of the family impact and needs of Dravet's syndrome in Spain. [Spanish]. Revista de Neurologia. 2020;70(3):75-83.

2. Aras LM, Isla J, Mingorance-Le Meur A. The European patient with Dravet syndrome: Results from a parent-reported survey on antiepileptic drug use in the European population with Dravet syndrome. Epilepsy and Behavior. 2015;44:104-9. <https://www.epilepsybehavior.com/article/S1525-5050(14)00698-2/pdf>

3. Auvin S, Damera V, Martin M, et al. The impact of seizure frequency on quality of life in patients with Lennox-Gastaut syndrome or Dravet syndrome. Epilepsy & Behavior. 2021;123:108239.

4. Bayat A, Hjalgrim H, Moller RS. The incidence of SCN1A-related Dravet syndrome in Denmark is 1:22,000: A population-based study from 2004 to 2009. Epilepsia. 2015;56(4):e36-e9.

5. Bjurulf B, Reilly C, Sigurdsson GV, et al. Dravet syndrome in children-A population-based study. Epilepsy Research. 2022;182:106922.

6. Brunklaus A, Dorris L, Zuberi SM. Comorbidities and predictors of health-related quality of life in Dravet syndrome. Epilepsia. 2011;52(8):1476-82.

7. Brunklaus A, Ellis R, Reavey E, et al. Prognostic, clinical and demographic features in SCN1A mutation-positive Dravet syndrome. Brain. 2012;135(8):2329-36.

8. Brunklaus A, Ghanty I, Dorris L, et al. Death in SCN1A positive Dravet syndrome-findings from a 9-year follow-up of 103 cases. Developmental Medicine and Child Neurology. 2019;61(Supplement 1):49-50.

9. Campbell JD, Whittington MD, Kim CH, et al. Assessing the impact of caring for a child with Dravet syndrome: Results of a caregiver survey. Epilepsy and Behavior. 2018;80:152-6. <https://www.epilepsybehavior.com/article/S1525-5050(17)30967-8/pdf>

10. Ceska K, Cesky L, Oslejskova H, et al. The Direct Costs of Dravet's Syndrome before and after Diagnosis Assessment. Neuropediatrics. 2021;52(1):6-11. <https://www.thieme-connect.com/products/ejournals/abstract/10.1055/s-0040-1718518>

11. Cooper MS, McIntosh A, Crompton DE, et al. Mortality in Dravet syndrome. Epilepsy Research. 2016;128:43-7.

12. de Lange IM, Gunning B, Sonsma AC, et al. Outcomes and comorbidities of SCN1A-related seizure disorders. Epilepsy & Behavior. 2019;90:252-9.

13. Gara-Adams R, Adams E, Mowlem F, et al. Care Pathway Mapping for Dravet Syndrome (DS) patients in England - Interim results from a pilot study. Epilepsia. 2021;62(SUPPL 3):169.

14. Genton P, Velizarova R, Dravet C. Dravet syndrome: The long-term outcome. Epilepsia. 2011;52(SUPPL. 2):44-9.

15. Hollenack K, Story T, Acs A, et al. Prevalence of probable dravet syndrome, lennox-gastaut syndrome, and other refractory epilepsies in commercial and medicaid populations in the United States. Journal of Managed Care and Specialty Pharmacy. 2019;25(3-A SUPPL.):S58.

16. Hollenack KA, Story TJ, Acs A, et al. Pnd34 Economic Burden of Probable Lennox-Gastaut Syndrome, Probable Dravet Syndrome, and Other Refractory Epilepsies for United States Medicaid Health Plans. Value in Health. 2019;22(Supplement 2):S276.

17. Huang CH, Hung PL, Fan PC, et al. Clinical spectrum and the comorbidities of Dravet syndrome in Taiwan and the possible molecular mechanisms. Scientific reports. 2021;11(1):20242.

18. Hurst DL. Epidemiology of severe myoclonic epilepsy of infancy. Epilepsia. 1990;31(4):397-400.

19. Lagae L, Brambilla I, Mingorance A, et al. Quality of life and comorbidities associated with Dravet syndrome severity: a multinational cohort survey. Developmental Medicine & Child Neurology. 2018;60(1):63-72.

20. Lagae L, Irwin J, Gibson E, et al. Caregiver impact and health service use in high and low severity Dravet syndrome: A multinational cohort study. Seizure. 2019;65:72-9. <https://www.seizure-journal.com/article/S1059-1311(18)30621-6/pdf>

21. Lo SH, Lloyd A, Marshall J, et al. Patient and Caregiver Health State Utilities in Lennox-Gastaut Syndrome and Dravet Syndrome. Clinical Therapeutics. 2021;43(11):1861-76.e16. <https://www.clinicaltherapeutics.com/article/S0149-2918(21)00390-8/pdf>

22. Nabbout R, Auvin S, Chiron C, et al. Development and content validation of a preliminary core set of patient- and caregiver-relevant outcomes for inclusion in a potential composite endpoint for Dravet Syndrome. Epilepsy and Behavior. 2018;78:232-42. <https://www.epilepsybehavior.com/article/S1525-5050(17)30357-8/pdf>

23. Nabbout R, Auvin S, Chiron C, et al. Perception of impact of Dravet syndrome on children and caregivers in multiple countries: looking beyond seizures. Developmental Medicine and Child Neurology. 2019;61(10):1229-36.

24. Nabbout R, Dirani M, Teng T, et al. Impact of childhood Dravet syndrome on care givers of patients with DS, a major impact on mothers. Epilepsy & Behavior. 2020;108:107094.

25. Nolan KJ, Camfield CS, Camfield PR. Coping with Dravet syndrome: Parental experiences with a catastrophic epilepsy. Developmental Medicine and Child Neurology. 2006;48(9):761-5.

26. Owen Pickrell W, Guelfucci F, Martin M, et al. Prevalence and healthcare resource utilization of patients with Dravet syndrome: Retrospective linkage cohort study. Seizure. 2022;99:159-63. <https://www.seizure-journal.com/article/S1059-1311(22)00122-4/pdf>

27. Pagano K, Irwin J, Lagae L, et al. The clinical and socioeconomic impact of Dravet syndrome in the UK. Developmental Medicine and Child Neurology. 2019;61(Supplement 1):62.

28. Paprocka J, Lewandowska A, Zielinski P, et al. Dravet Syndrome-The Polish Family's Perspective Study. Journal of Clinical Medicine. 2021;10(9):28.

29. Reaven NL, Funk SE, Lyons PD, et al. The direct cost of seizure events in severe childhood-onset epilepsies: A retrospective claims-based analysis. Epilepsy and Behavior. 2019;93:65-72. <https://www.epilepsybehavior.com/article/S1525-5050(18)30907-7/pdf>

30. Rosander C, Hallbook T. Dravet syndrome in Sweden: a population-based study. Developmental Medicine & Child Neurology. 2015;57(7):628-33.

31. Sakauchi M, Oguni H, Kato I, et al. Retrospective multiinstitutional study of the prevalence of early death in Dravet syndrome. Epilepsia. 2011;52(6):1144-9.

32. Sakauchi M, Oguni H, Kato I, et al. Mortality in Dravet syndrome: Search for risk factors in Japanese patients. Epilepsia. 2011;52(SUPPL. 2):50-4.

33. Schoonjans AS, De Keersmaecker S, Van Bouwel M, et al. More daytime sleepiness and worse quality of sleep in patients with Dravet Syndrome compared to other epilepsy patients. European Journal of Paediatric Neurology. 2019;23(1):61-9.

34. Schubert-Bast S, Kay L, Simon A, et al. Epidemiology, healthcare resource use, and mortality in patients with probable Dravet syndrome: A population-based study on German health insurance data. Epilepsy and Behavior. 2022;126 (no pagination).

35. Sinoo C, de Lange IM, Westers P, et al. Behavior problems and health-related quality of life in Dravet syndrome. Epilepsy & Behavior. 2019;90:217-27.

36. Skluzacek JV, Watts KP, Parsy O, et al. Dravet syndrome and parent associations: The IDEA League experience with comorbid conditions, mortality, management, adaptation, and grief. Epilepsia. 2011;52(SUPPL. 2):95-101.

37. Steckler F, Dunwoody B, Dorris L, et al. Cognition and disease burden in SCN1A positive Dravet syndrome-a 10-year follow-up study. Developmental Medicine and Child Neurology. 2020;62(Supplement 1):24-5.

38. Stockl KM, Hollenack KA, Acs A, et al. Pnd27 Economic Burden of Probable Lennox-Gastaut Syndrome, Probable Dravet Syndrome, and Other Refractory Epilepsies for United States Commercial Health Plans. Value in Health. 2019;22(Supplement 2):S274.

39. Stockl K, Funk S, Reaven N, et al. Inpatient hospitalizations and readmissions among patients with probable lennox-gastaut syndrome, dravet syndrome, tuberous sclerosis complex, and other refractory epilepsies. Journal of Managed Care and Specialty Pharmacy. 2019;25(10-A SUPPL.):S57-S8.

40. Strzelczyk A, Schubert-Bast S, Reese JP, et al. Evaluation of health-care utilization in patients with Dravet syndrome and on adjunctive treatment with stiripentol and clobazam. Epilepsy and Behavior. 2014;34:86-91. <https://www.epilepsybehavior.com/article/S1525-5050(14)00095-X/fulltext>

41. Strzelczyk A, Kalski M, Bast T, et al. Burden-of-illness and cost-driving factors in Dravet syndrome patients and carers: A prospective, multicenter study from Germany. European Journal of Paediatric Neurology. 2019;23(3):392-403. <https://www.ejpn-journal.com/article/S1090-3798(18)30542-7/pdf>

42. Strzelczyk A, Schubert-Bast S, Bast T, et al. A multicenter, matched case-control analysis comparing burden-of-illness in Dravet syndrome to refractory epilepsy and seizure remission in patients and caregivers in Germany. Epilepsia. 2019;60(8):1697-710. <https://onlinelibrary.wiley.com/doi/pdfdirect/10.1111/epi.16099?download=true>

43. Strzelczyk A, Lagae L, Kurlemann G, et al. Clinical characteristics and quality of life with Dravet syndrome: results of the German cohort of the Dravet syndrome caregiver survey (DISCUSS). [German]. Zeitschrift fur Epileptologie. 2022;35(2):169-77.

44. Strzelczyk A, Kurlemann G, Bast T, et al. Exploring the relationships between composite scores of disease severity, seizure-freedom and quality of life in Dravet syndrome. Neurological Research & Practice. 2022;4(1):22.

45. Symonds JD, Dorris L, Brunklaus A, et al. Epidemiology of early childhood epilepsies: Syndrome classification and association with socioeconomic status. Developmental Medicine and Child Neurology. 2021;63(SUPPL 1):6-7.

46. Umeno J. A survey on the utilization status of social welfare services for pediatric patients with epilepsy. [Japanese]. No To Hattatsu. 2019;51(4):234-9.

47. Villas N, Meskis MA, Goodliffe S. Dravet syndrome: Characteristics, comorbidities, and caregiver concerns. Epilepsy and Behavior. 2017;74:81-6. <https://www.epilepsybehavior.com/article/S1525-5050(17)30351-7/pdf>

48. Whittington MD, Knupp KG, Vanderveen G, et al. The direct and indirect costs of Dravet Syndrome. Epilepsy and Behavior. 2018;80:109-13. <https://www.epilepsybehavior.com/article/S1525-5050(17)30934-4/fulltext>

49. Wu YW, Sullivan J, McDaniel SS, et al. Incidence of Dravet Syndrome in a US Population. Pediatrics. 2015;136(5):e1310-5. <https://www.ncbi.nlm.nih.gov/pmc/articles/PMC4621800/pdf/peds.2015-1807.pdf>

50. Hoy D, Brooks P, Woolf A, et al. Assessing risk of bias in prevalence studies: modification of an existing tool and evidence of interrater agreement. J Clin Epidemiol. 2012;65(9):934-9. 10.1016/j.jclinepi.2011.11.014

51. Gallop K, Lloyd AJ, Olt J, et al. Impact of developmental and epileptic encephalopathies on caregivers: A literature review. Epilepsy & Behavior. 2021;124:108324. <https://doi.org/10.1016/j.yebeh.2021.108324>

52. Molinier L, Bauvin E, Combescure C, et al. Methodological considerations in cost of prostate cancer studies: a systematic review. Value Health. 2008;11(5):878-85. 10.1111/j.1524-4733.2008.00327.x
